# Chromatin Modulatory Proteins and Olfactory Receptor Signaling in the Refinement and Maintenance of *Fruitless* Expression in Olfactory Receptor Neurons

**DOI:** 10.1371/journal.pbio.1002443

**Published:** 2016-04-19

**Authors:** Catherine E. Hueston, Douglas Olsen, Qingyun Li, Sumie Okuwa, Bo Peng, Jianni Wu, Pelin Cayirlioglu Volkan

**Affiliations:** 1 Department of Neurobiology, Duke University, Durham, North Carolina, United States of America; 2 Department of Biology, Duke University, Durham, North Carolina, United States of America; 3 Undergraduate Program in Neuroscience, Duke University, Durham, North Carolina, United States of America; 4 Duke Institute for Brain Science, Duke University, Durham, North Carolina, United States of America; Vlaams Instituut voor Biotechnologie and Katholieke Universiteit Leuven, BELGIUM

## Abstract

During development, sensory neurons must choose identities that allow them to detect specific signals and connect with appropriate target neurons. Ultimately, these sensory neurons will successfully integrate into appropriate neural circuits to generate defined motor outputs, or behavior. This integration requires a developmental coordination between the identity of the neuron and the identity of the circuit. The mechanisms that underlie this coordination are currently unknown. Here, we describe two modes of regulation that coordinate the sensory identities of *Drosophila melanogaster* olfactory receptor neurons (ORNs) involved in sex-specific behaviors with the sex-specific behavioral circuit identity marker *fruitless* (*fru*). The first mode involves a developmental program that coordinately restricts to appropriate ORNs the expression of *fru* and two olfactory receptors (Or47b and Ir84a) involved in sex-specific behaviors. This regulation requires the chromatin modulatory protein Alhambra (Alh). The second mode relies on the signaling from the olfactory receptors through CamK and histone acetyl transferase p300/CBP to maintain ORN-specific *fru* expression. Our results highlight two feed-forward regulatory mechanisms with both developmentally hardwired and olfactory receptor activity-dependent components that establish and maintain *fru* expression in ORNs. Such a dual mechanism of *fru* regulation in ORNs might be a trait of neurons driving plastic aspects of sex-specific behaviors.

## Abstract 2

The *fruitless* (*fru*) gene regulates sexual behavior in flies, but what regulates *fru*? This study shows that the chromatin modulator Alhambra restricts *fru* expression to specific neurons in the developing *Drosophila* olfactory system. In adults, olfactory receptor signaling and the histone acetyltransferase p300 are required to maintain *fru* expression.

## Author Summary

How do individual neurons know what type of a circuit they must integrate into? To correctly assemble neural circuits during development, the identities of neurons must be coordinated with the identities of the circuits into which they will be integrated. How is this process regulated? We have used the olfactory circuits that regulate sex-specific behaviors in *Drosophila* to answer this question. In *Drosophila*, the *fruitless* (*fru*) gene, which encodes a transcription factor, acts as a molecular marker that labels circuits regulating sex-specific behaviors. *Fru* is both a necessary and sufficient regulator of sex-specific behaviors like courtship and aggression and is expressed in only about 2,000 interconnected neurons in the fly nervous system. Even though *fru* expression and function is so critical to sex-specific behavior, the mechanisms regulating its expression in each of the neurons within the circuit are not known. Here, we revealed two different modes of transcriptional regulation of *fru* during the development of the olfactory receptor neurons that are involved in sex-specific behaviors. In the first mode, the putative chromatin modulator Alhambra (Alh) coregulates both olfactory receptors and *fru* expression in two olfactory receptor neuron classes during development. In adult flies, the second mode maintains *fru* expression through the activity of olfactory receptors, calcium signaling, and chromatin modulatory proteins in these olfactory receptor neurons. These two genetic programs separate the developmentally hardwired and the olfactory receptor activity-mediated regulation of *fru* expression in olfactory circuits, and might represent the molecular mechanisms that mediate the innate and adaptable aspects of odor-guided social behaviors.

## Introduction

The assembly of neural circuits dedicated to specific behaviors must be tightly regulated during development, where neurons need not only define their identity as individual neurons but also molecularly and developmentally link themselves with the specific neural circuitry they will be integrated into. Such developmental programs can, for example, establish a connection between sensory circuits tuned to a particular stimulus and the motor pathways that execute the output behavior in response to that stimulus. An elegant example of this is seen in the sex-specific behavioral circuitry of *Drosophila melanogaster*. This circuitry is regulated by the single transcription factor Fruitless (Fru) [1]. Sex-specific alternative splicing *of fru* generates the protein product Fru^M^ in males only [2–5]. Studies in which the sex-specific splicing of *fru* was manipulated in both males and females have shown that Fru^M^ function is necessary and sufficient for male-specific behaviors [6]. Despite the dramatic nature of these mutant phenotypes, *fru* is only expressed in a small fraction of the *D*. *melanogaster* nervous system. Only about 2,000 interconnected neurons express *fru*, and the specific activation of *fru*-positive neurons is sufficient to trigger male-specific behaviors [1,2,6–8]. Thus, *fru* expression identifies the neural circuitry that controls sex-specific behaviors. Support for this idea comes from the recently identified *fru*-positive neuronal circuitry that drives the sexually dimorphic response to the pheromone cis-vaccenyl acetate (cVA) [9]. *fru* is expressed throughout the entire cVA circuitry, from the olfactory receptor neurons (ORNs) that detect the pheromone to the motor neurons that trigger courtship behaviors [10,11]. Despite a large volume of research, it is largely unknown how Fru regulates the development or function of this circuitry and how *fru* expression is developmentally coordinated with the identity programs for each of the neurons within the circuitry.

Olfaction is a key component of sex-specific behaviors. Flies detect volatile pheromones and other odors important for the initiation of courtship and aggression via olfactory receptors (ORs) expressed in ORNs [12]. Clusters of 1–4 ORNs are housed in sensory hairs called sensilla that cover the surface of the antennae and maxillary palps. ORNs are classified and named by the single OR gene that they express, and each ORN class targets a specific glomerulus in the antennal lobe. Fru^M^ function was shown to be specifically required in ORNs for normal courtship behavior [2], and three ORN classes express *fru* in adult *D*. *melanogaster* antennae [12]. Or67d ORNs, the best studied of the *fru*-positive ORNs, are housed in the at1 sensilla. These neurons detect the male-specific pheromone cVA, which acts as a suppressant of both male—male and male—female courtship [9]. Ir84a ORNs, housed in the ac4 sensilla, also express *fru* and can detect the availability of food sources and coordinate reproductive behaviors accordingly [13]. Recent studies show that the third *fru*-positive ORN class, Or47b, detects methyl laurate, a cuticular pheromone, and is necessary for successful copulation [14–17].

Neurons expressing *Or67d*, *Ir84a*, and *Or47b* all express *fru* and are specifically receptive to olfactory cues required for courtship, but the developmental programs regulating the expression of these three *Or* genes and coordinating them with *fru* expression is unknown. During development, combinations of transcription factors diversify ORN precursor cell identities, thus restricting the ORN classes that can be generated in each precursor lineage [18,19]. This “cellular memory” of possible fates is retained through asymmetric divisions as Notch signaling further segregates each possible sensory identity into individual ORNs within the same sensillum [12,20,21]. The retention of cellular identity through multiple cell divisions suggests that changes in chromatin states might also contribute to these programs [22,23]. We speculate that existing OR regulation can be co-opted for coregulation of *fru* expression in ORNs with sex-specific behavioral functions.

Here, we describe a molecular circuitry with both developmentally hardwired and olfactory-receptor-activity-dependent components that refine and maintain *fru* expression in Or47b and Ir84a ORNs. In a genetic screen for ORN development, we identified a putative chromatin modulator, Alhambra/AF10 (Alh), which, when mutated, expands both *fru/Or47b* and *fru/Ir84a* expression to *fru*-negative ORNs independently of axon guidance decisions during development. Alh is expressed dynamically in developing ORNs in pupal stages, but during the onset of OR expression it is expressed in ORNs that do not express *fru*. Once the correct pattern of OR and *fru* expression is established, the maintenance of *fru* expression requires Or47b and Ir84a activity in adult flies. This mode of *fru* regulation in adult ORNs is disrupted in CamKI and histone acetyl transferase p300/CREB Binding Protein (CBP) mutants. Our results suggest that Alh and OR-dependent signaling represent two different modes of *fru* transcriptional regulation that coordinate, establish, and maintain the *fru*-positive identity alongside the sensory identities of ORNs.

## Results

### *fru*-Positive OR Expression Is Expanded to *fru*-Negative ORNs in *p* ^*1353*^ Mutants

**Fig 1:**
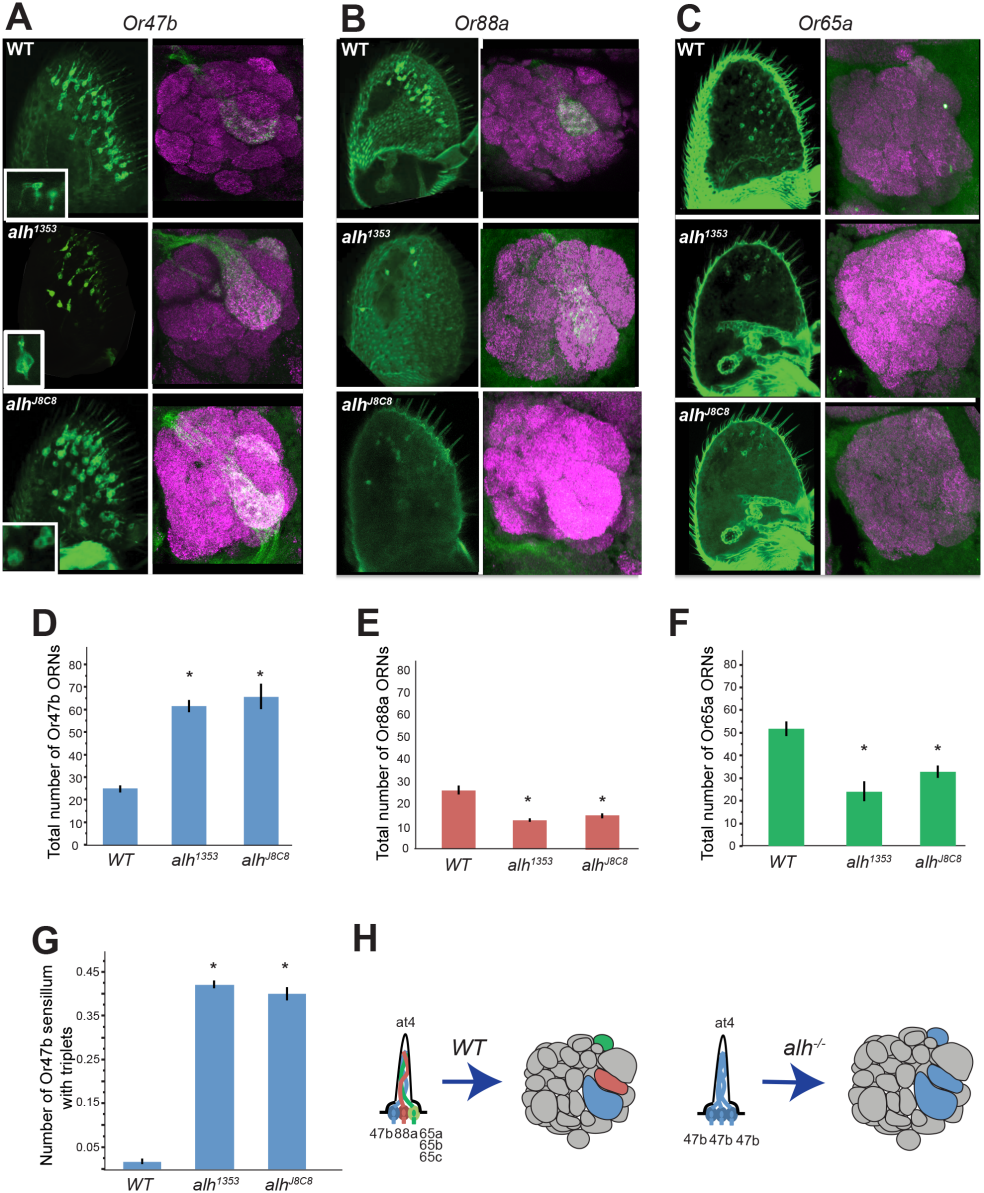
*fru*-positive OR expression in at4 sensilla expands to developmentally related *fru*-negative ORNs in *alh* mutants. ** A)** Adult antennae and brains labeled with *Or47bGal4 UAS-CD8GFP* (green) in wild type and *alh* mutant clones. Magenta staining in brains is against N-cadherin, a neuropil marker. **B)** Adult antennae and brains labeled with *Or88aGal4 UAS-CD8GFP* in wild-type and *alh* mutant clones. **C)** Adult antennae and brains labeled with *Or65aGal4 UAS-CD8GFP* in wild-type and *alh* mutant clones. **D–G)** Quantification of cell bodies observed in the adult antennae of WT and *alh* mutant clones. For all graphs, asterisks indicate significant (*p* < .05) differences from wild type. Error bars represent standard error of the mean (SEM). An ANOVA was performed for each cell type and followed with Tukey’s Honest Significant Difference (HSD)—see Materials and Methods. **D)** Total *Or47b*-positive cells. Wild type flies were significantly different from both *alh* conditions (*p* < .0001). *n* = 10–40. All count data may be found in the Supporting Information as S1 Data. **E)** Total *Or88a*-positive cells. Both *alh* conditions were significantly different from wild-type males (*p* < .0001). *n* = 27 − 57. **F)** Total *Or65a*-positive cells. Both *alh* conditions were significantly different from wild-type males (*p* < .05). *n* = 9–27. **G)** Total *or47b*-positive clusters, normalized by total *Or47b*-positive cells. Wild type flies were significantly different from all *alh* conditions (*p* < .0001). *n* = 10–40. **H)** Model: in *alh* mutants, the *Or47b* odorant receptor expression is expanded to the other ORNs in the at4 sensilla, at the expense of their native OR expression, but the axons of these ORNs continue to target their original locations in the antennal lobe. GENOTYPES: A) *eyflp*; *Or47bGal4/UAS-CD8GFP*; *FRT82/FRT82Gal80E2F*, *eyflp*; *Or47bGal4*/*UAS-CD8GFP*; *FRT82alh* ^*1353*^/*FRT82Gal80E2F*, *eyflp*; *Or47bGal4*/*UAS-CD8GFP*; *FRT82alh* ^*j8c8*^/*FRT82Gal80E2F* B) *eyflp*; *Or88aGal4/UAS-CD8GFP*; *FRT82/FRT82Gal80E2F*, *eyflp*; *Or88aGal4*/*UAS-CD8GFP*; *FRT82alh* ^*1353*^/*FRT82Gal80E2F*, *eyflp*; *Or88aGal4*/*UAS-CD8GFP*; *FRT82alh* ^*j8c8*^/*FRT82Gal80E2F* C) *eyflp*; *Or65aGal4/UAS-CD8GFP*; *FRT82/FRT82Gal80E2F*, *eyflp*; *Or65aGal4*/*UAS-CD8GFP*; *FRT82alh* ^*1353*^/*FRT82Gal80E2F*, *eyflp*; *Or65aGal4*/*UAS-CD8GFP*; *FRT82alh* ^*j8c8*^/*FRT82Gal80E2F*

We previously carried out a forward genetic screen for regulators of class-specific ORN development in the olfactory system [24]. This was a histology-based mutagenesis screen where the glomerular targeting patterns of three different classes of ORNs (Or47a, Or47b, and Gr21a) were analyzed in antennal mutant clones in an otherwise heterozygous animal [25]. We specifically looked for mutants with defective ORN projection patterns in the antennal lobe. In this screen, we isolated a mutation (*p* ^*1353*^) that modified the projection pattern of Or47b ORNs in the antennal lobe (Fig 1). We mapped this mutation to the *D*. *melanogaster* orthologue of the chromatin modulatory protein AF10, *Alhambra (alh)*. We refer to this mutation as *alh* ^*1353*^ in the rest of the paper. In both *alh* ^*1353*^ and *alh* ^*J8C8*^ mutants, Or47b ORN axons project to their normal target, the VA1v glomerulus, as well as the DL3 glomerulus (Fig 1A and 1H). In addition, the VA1v glomerulus expands dorsally, appearing to almost engulf the VA1d glomerulus, leading to a loss of VA1d glomerulus in some of the mutant antennal lobes [26]. The DL3 and VA1d glomeruli are normally innervated by other at4 sensilla ORNs that express *Or65a* and *Or88a*, respectively, which develop from the same sensory organ precursor as Or47b ORNs [20].

In *D*. *melanogaster*, ORNs housed in the same sensillum arise through asymmetric divisions of a single multipotent precursor cells. During these divisions, the axons from the ORNs sort themselves and navigate to different future glomerular regions in the antennal lobe. Unlike mammals, ORs in *D*. *melanogaster* are not required for ORN axon guidance [27,28]. In fact, axon sorting decisions that guide ORNs from the same sensillum to distinct glomeruli are made prior to OR expression, suggesting that independent programs regulate the sensory identities and guidance of ORN axons [29]. The defects in the glomerular pattern of Or47b ORN projections in *alh* mutants could be due to a disruption of developmental programs regulating Or47b ORN axon guidance. Alternatively, the same defects might arise due to a conversion of Or65a and Or88a ORNs to the Or47b sensory identity without affecting the glomerular position of the at4 ORNs. In order to differentiate between these two hypotheses, we analyzed *Or47b* expression in the antenna. In wild type antennal MARCM clones [30], one Or47b ORN is found in each at4 sensillum. However, in *alh* ^*1353*^ mutant clones, *Or47b* expression is expanded within the same sensillum, leading to 2–3 Or47b ORNs per sensillum, all of which extend their dendrites into the same sensory bristle (Fig 1A). This was verified by counting the total number of cells in each mutant antenna, a somewhat variable number due to differences in the sizes of the clones produced by MARCM (Fig 1D). To reduce this variability, we also quantified the number of clusters of cells (defined as cell bodies with dendrites that project to the same sensillum) and divided this number by the total number of cells to normalize by the size of the clonal population (Fig 1G). Examination of *Or88a* and *Or65a* expression showed a concomitant decrease in the number of Or88a and Or65a ORNs, suggesting a conversion of Or65a and Or88a to Or47b sensory identity (Fig 1B–1F). These changes in *Or47b*, *Or88a*, and *Or65a* expression were also confirmed by qRT-PCR of wild type and *alh* mutant antennae (S1 Fig). In addition, double-labeling experiments show that the few remaining Or88a ORNs in *alh* mutants are never found in “clusters” in the antennae, and their axons intermingle with the converted Or47b axons in the VA1d glomerulus (S4A Fig).

We also overexpressed different isoforms of Alh. We hypothesized that if Alh is required to suppress Or88a and activate Or47b fate, overexpression of Alh should result in the decrease of Or47b ORNs and expansion of Or88a fate. Overexpression of the long isoform of *alh*, *alh-L*, using *elav-GAL4*, did not result in any Or88a or Or47b phenotype (S5A Fig). *Elav-GAL4*-induced expression of *alh-S* was lethal, suggesting this is the functionally important isoform. We then used heatshock-induced expression of GAL4 to pulse *alh-S* expression through third instar-10 h after pupanium formation (APF), 10–30 h APF, 24–34 h APF, and 48–58 h APF old pupae. None of these experiments resulted in an expanded Or88a or lost Or47b ORN population (S5 Fig). These results suggest that OR expression in at4 ORNs is more sensitive to loss of *alh* function rather than overexpression. One possible explanation for this can be that overexpression of *alh* might not be strong enough to overcome lineage-specific chromatin states around at4 OR genes that are already in place during at4 ORN development at the times of our heatshock. In addition, it is still possible that, due to the highly dynamic expression pattern of *alh*, there is a very narrow time window during pupal stages that was not captured in our heatshock experiments. Thus, we base our interpretations on the results from the mutant analysis, which suggests a function for Alh in appropriate segregation of ORs identities of ORNs independent of their guidance decisions to specific glomerular zones, uncoupling the genetic programs for sensory receptor selection and axon guidance.

**Fig 2:**
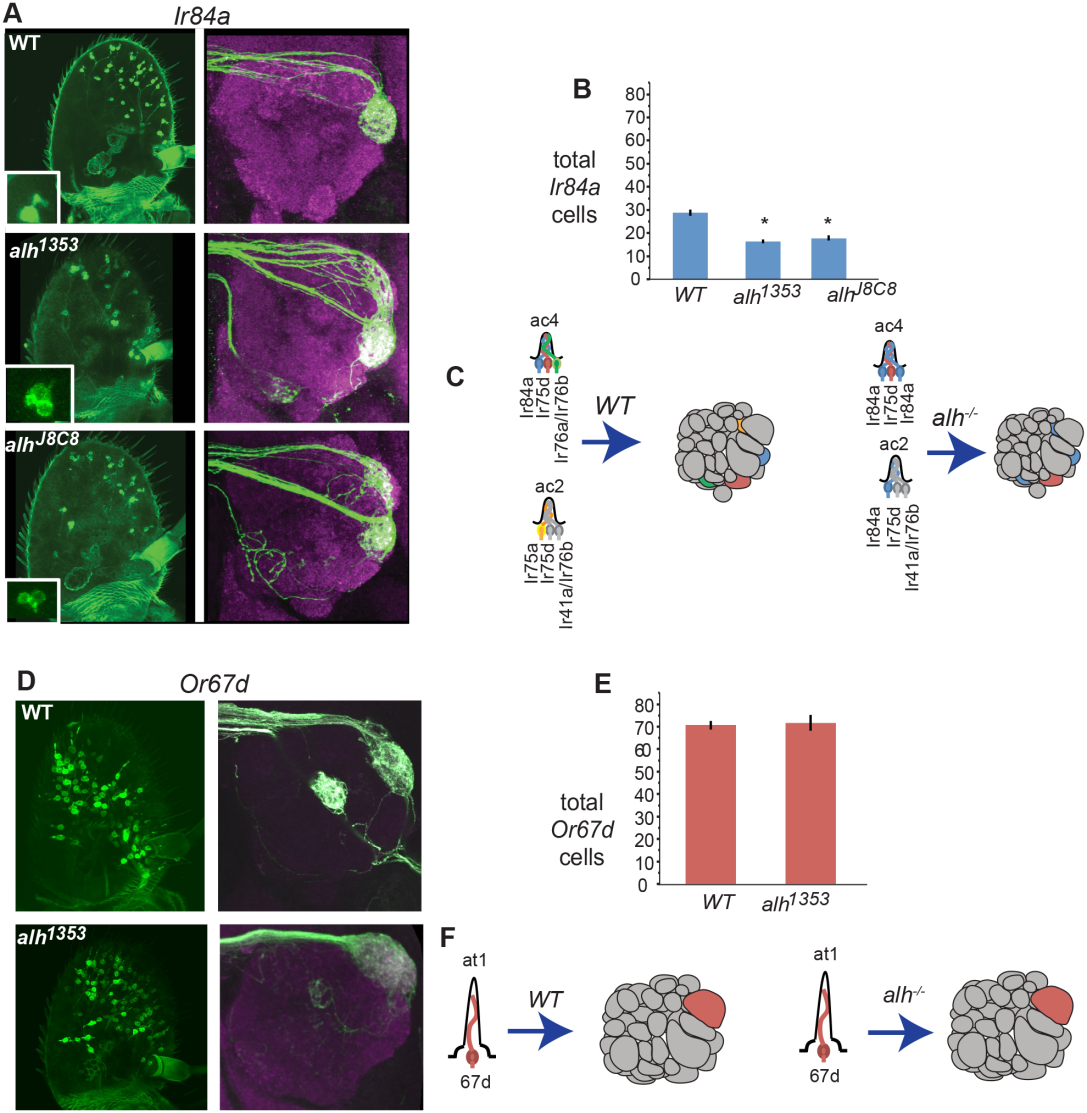
*fru*-positive OR expression in ac4 sensilla expands to developmentally related *fru*-negative ORNs in *alh* mutants. **A)** Adult antennae and brains labeled with *Ir84aGal4 UAS-CD8GFP* (green) in wild type and *alh* mutant clones. Magenta staining in brains is against N-cadherin, a neuropil marker. **B)** Total *Ir84a*-positive cells. Asterisks indicate significant (*p* < .05) differences from wild type. Error bars represent SEM. ANOVAs were performed and followed with Tukey’s HSD—see Materials and Methods. Wild type flies were significantly different from all *alh* conditions (*p* < .0001). *n* = 24–50. All count data may be found in the Supporting Information as S1 Data. **C) Model:** In *alh* mutants, the *Ir84a* odorant receptor identity is expanded to other coeloconic ORNs as observed through glomerular innervation. *Ir84a* expression is expanded to *ir75a* and *ir76a* ORNs. **D)** Adult antennae and brains labeled with *Or67dGal4 UAS-CD8GFP* (green) in wild type and *alh* mutant clones in *Drosophila*. Magenta staining in brains is against N-cadherin, a neuropil marker. **E)** Total *Or67d*-positive cells. An ANOVA for this data was not significant. *n* = 20–30. All count data may be found in the Supporting Information as S1 Data. **F)** Model: In *alh* mutants, the expression and axonal targeting patterns of *or67d*-positive ORNs are unchanged. GENOTYPES: A) *eyflp*; *Ir84aGal4/UAS-CD8GFP*; *FRT82/FRT82Gal80E2F*, *eyflp*; *Ir84aGal4*/*UAS-CD8GFP*; *FRT82alh* ^*1353*^/*FRT82Gal80E2F*, *eyflp*; *Ir84aGal4*/*UAS-CD8GFP*; *FRT82alh* ^*j8c8*^/*FRT82Gal80E2F* D) *eyflp*; *Or67dGal4/UAS-CD8GFP*; *FRT82/FRT82Gal80E2F*, *eyflp*; *Or67dGal4*/*UAS-CD8GFP*; *FRT82alh* ^*1353*^/*FRT82Gal80E2F*

The majority of the other ORN classes representing approximately ten different sensilla, including Or67d ORNs in at1 sensilla (Fig 2D–2F) examined, were not affected by similar sensory conversions (S2 Fig). In contrast, we detected a similar identity conversion in the sensilla housing Ir84a ORNs (Fig 2A–2C). We detected a general decrease in the total number of ac4 sensilla based on cell counts in *alh* ^*1353*^ mutants (Fig 2B). This possibly is due to an earlier function of Alh on precursor patterning based on its expression in a ring of cells on the antennal disc known to give rise to some of the coeloconic and trichoid sensilla fates [31] (S6 Fig), which in *alh* mutants lead to decreased cell survival. Despite the effects on the total number of sensilla, within the formed sensilla, multiple *Ir84a*-positive ORNs were observed (Fig 2A–2C).

Each Ir84a ORN is normally housed in the ac4 sensilla with its sibling ORNs that express *Ir75d* and *Ir76a* (Fig 2C) [31]. Examination of the antennal lobes of *alh* ^*1353*^ mutants showed that in addition to the VL2a glomerulus normally innervated by Ir84a ORNs, *Ir84a*-positive ORNs were also present in the VM4 glomerulus of Ir76a ORNs (Fig 2A and 2C) [32]. We found that *Ir76a* expression was decreased in *alh* mutants (S4C Fig). We also observed *Ir84a*–positive ORN terminals innervating the DP1l glomerulus housing Ir75a ORNs, which normally reside in ac2 and ac3 sensilla, suggesting that ectopic expression of *Ir84a* is not restricted to the ac4 ORNs but also expands to other coeloconic sensilla ORNs. From our previous studies, we know that ORN organization and development in coeloconic sensilla is not identical to ORNs in basiconic and trichoid sensilla [18,31,32]. For example, ac2, ac3, and ac4 sensilla all share the same neurons, Ir76b, Or75d, Ir75a and Or76b, in different combinations, and thus they are more developmentally intertwined than other sensilla types, which does not happen in basiconic and trichoid sensilla. It is possible that the differences of *Or47b* and the *Ir84a* expression phenotypes in *alh* ^*1353*^ mutants might reflect differences in the developmental programs of trichoid (at4) versus coeloconic (ac4) sensilla. In summary, our results suggest that in *alh* ^*1353*^ mutants, two *fru*-positive ORs (*Or47b* and *Ir84a*) expand to developmentally related ORNs without changing the projection of their axons to appropriate glomerular areas in the antennal lobe.

### *Fru* Expression Accompanies Sensory Conversion in *alh* ^*1353*^ Mutants

Our results demonstrate that Alh regulates some aspects of ORN identity, such as *Or* expression, but not others, such as axon sorting and guidance. We then asked what other aspects of ORN identity might be regulated by *alh* ^*1353*^. We were intrigued that the two ORNs expanded by the *alh* ^*1353*^ mutation, Or47b and Ir84a, are two of the three ORN classes that express *fru*. To answer whether other aspects of neuronal identity are also altered in *alh* ^*1353*^ mutants, we examined *fru* expression.

**Fig 3:**
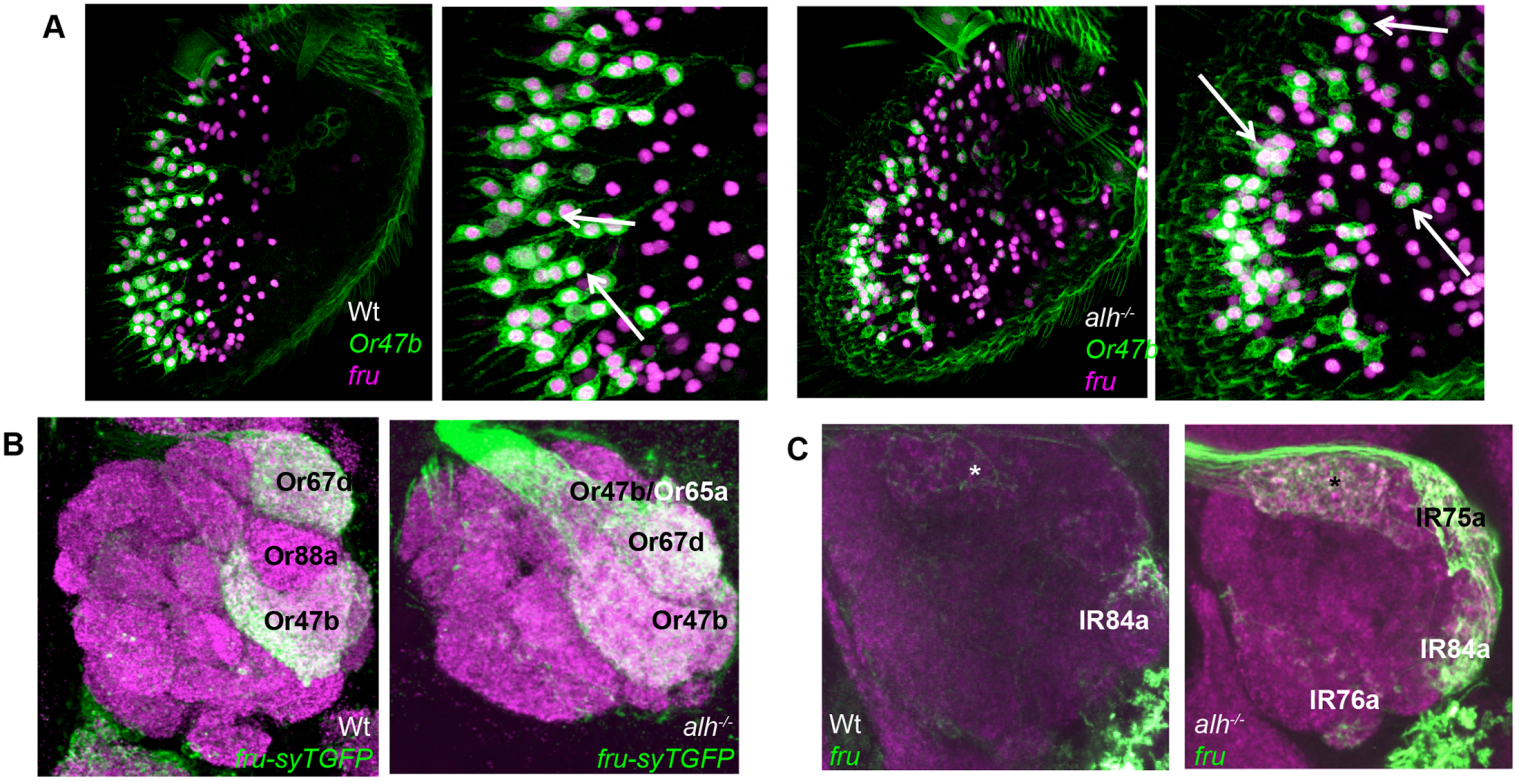
*fru* expression expands together with *Or47b* expression in *alh* mutants. **(A/B)** Antennae labeled with *fru* ^*GAL4*^ *UAS-RedStinger* (magenta), and *Or47bCD8GFP* (green) in wild type and *alh* mutant clones. Right panels represent higher magnification images. Arrows label *Or47b/fru*-positive nuclei in wild type images. In *alh* mutants, arrows point to sensilla with 2–3 Or47b ORNs that are also *fru*-positive. **(C)** Antennal lobes labeled with fruGal4 UAS-sytGFP (Z-stack, anterior sections of antennal lobe). **(D)** Antennal lobes labeled with fruGal4 UAS-CD8GFP (Z-stack, posterior sections of antennal lobe). Asterisks denote *fru*-labeled glomeruli thought to be innervated by neurons from the antennal sacculus. GENOTYPES: (A) wild type: *eyFLP/+;Or47bCD8GFP/UAS-RedStinger; FRT82 fru* ^*GAL4*^ */FRT82Gal80E2F* (B) *alh* mutant: *eyFLP/+;Or47bCD8GFP/UAS-RedStinger; FRT82 alh* ^*1353*^ *fru* ^*GAL4*^ */FRT82Gal80E2F* (C) wild type: *eyFLP/+; UAS-syTGFP/+; FRT82 fru* ^*GAL4*^ */FRT82Gal80E2F* *alh* mutant: *eyFLP/+; UAS-syTGFP/+; FRT82 alh* ^*1353*^ *fru* ^*GAL4*^ */FRT82Gal80E2F* (D) wild type: *eyFLP/+; UAS-CD8GFP/+; FRT82 fru* ^*GAL4*^ */FRT82Gal80E2F* *alh* mutant: *eyFLP/+; UAS-CD8GFP/+; FRT82 alh* ^*1353*^ *fru* ^*GAL4*^ */FRT82Gal80E2F*

We observed an expansion of *fru* expression in *alh* ^*1353*^ mutant antennae (Fig 3). When we examined *fru* expression closely in *alh* ^*1353*^ mutant ORNs, we found clusters of 2–3 *fru*-positive ORNs within the same sensillum, mimicking *Or47b* expression in the *alh* ^*1353*^ mutants (Fig 3B). Indeed, colabeling for *Or47b* and *fru* revealed that both were coexpressed in *alh* mutant ORN clusters (Fig 3B). The expansion of *fru* expression to multiple at4 and ac4 ORNs observed in the antenna was confirmed when we examined *fru*-positive axon guidance in the antennal lobes of *alh* ^*1353*^ mutants, which show a striking similarity to the expression patterns of Or47b and Ir84a in *alh* ^*1353*^ mutants. (Fig 3C and 3D). Additional double-labeling experiments show that the innervation of the Va1d glomerulus by *fru*-positive axons in *alh* mutants is a result of the expansion of Or47b- and *fru*-positive axons to this location as previously found. The remaining nonclonal wild type Or88a ORNs in these mutants do not express *fruitless* (S4B Fig). These results suggest that *fru* expression accompanies *Or47b* and *Ir84a* expansion, and *alh* ^*1353*^ disrupts a program that is normally required to corepress both *Or47b/Ir84a* and *fru* expression in inappropriate, yet developmentally related ORNs.

### *alh*-Dependent Regulation of *Or* and *fru* Expression Occurs Early in *ORN* Development

**Fig 4:**
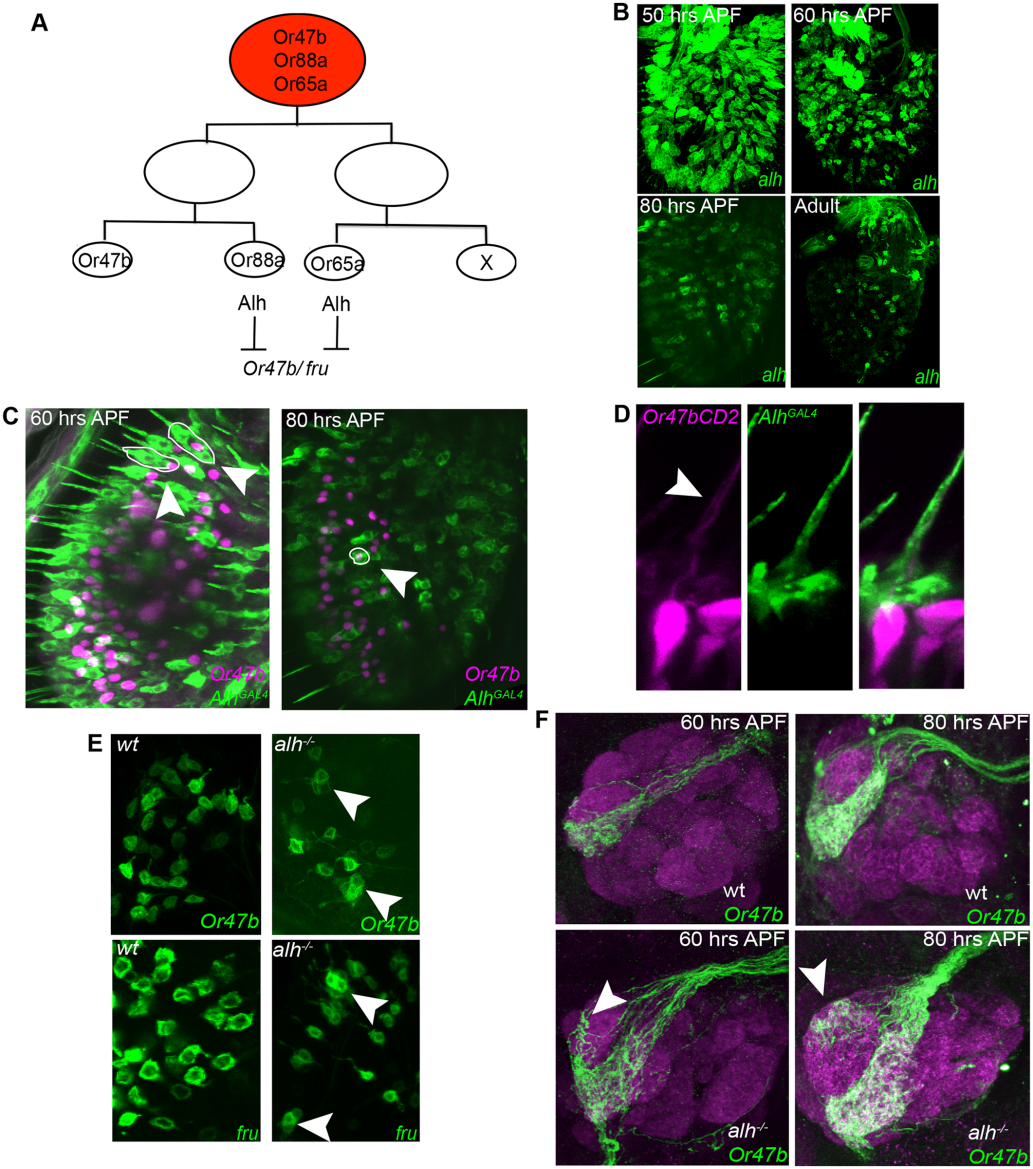
Alh represses *Or47b* and *fru* in developmentally related ORNs in the same sensillum during development. **(A)** Asymmetric divisions of neuronal precursors give rise to ORNs in at4 sensilla. From the mutant phenotype, we predict that Alh represses *Or47b* and *fru* expression in Or88a and Or65a ORNs. **(B)** *Alh* ^*GAL4*^-driven UAS-CD8GFP expression in developing pupal antennae. **(C)** Double labeling of *Or47b* (magenta) and *alh* (green) around 60–70 h APF (left panel) and 80 h APF. *Or47b* expression is excluded from *alh* expressing cells (arrows) but clusters with them (circled in white). **(D)** High magnification of a sensillum double labeled with *Or47b-CD2* (magenta) and *alh* (green). Arrow points to the dendrite of Or47b ORN innervating the sensory hair together with *alh*-positive fibers. **(E)** Enlarged portions of wild-type (left panels) and *alh* mutant (right panels) antennae at 60–70 h APF, expressing Or47b-Gal4 UASCD8GFP (top panels), or fru-gal4 UASCD8GFP (bottom panels). **(F)** Wild-type (top panels) and *alh* mutant (bottom panels) antennal lobes expressing Or47bGal4UASCD8GFP (green) and stained for *ncadherin* (magenta). GENOTYPES: (B) 50hrs: *AlhGal4* ^*NP7010*^/*UAS-CD8GFP*; 60hrs-adult: *AlhGal4* ^*NP7441*^/*UAS-CD8GFP* (C) *Or47b-lexA lexOp-tomato*:*nls AlhGal4* ^*NP6628*^/*UAS-CD8GFP* (D) *Or47B-CD2/+; AlhGal4* ^*NP6628*^/*UAS-CD8GFP* (E) *eyFLP/+; Or47b-GAL4 UAS-CD8GFP/+; FRT82/FRT82Gal80E2F* *eyFLP/+*; *Or47b-GAL4 UAS-CD8GFP/+; FRT82 alh^1353^ /FRT82Gal80E2F* or *eyFLP/+; UAS-CD8GFP/+; FRT82 fru* ^*GAL4*^ */FRT82Gal80E2F* *eyFLP/+*; *UAS-CD8GFP/+; FRT82 alh^1353^ fru^GAL4^/FRT82Gal80E2F* (F) *eyFLP/+; Or47b-GAL4 UAS-CD8GFP/+; FRT82 /FRT82Gal80E2F* or *eyFLP/+; Or47b-GAL4 UAS-CD8GFP/+; FRT82 alh* ^*1353*^ */FRT82Gal80E2F*

We hypothesized that in wild-type flies Alh, either directly or indirectly, coordinates repression of *fru* and *Or* expression in *fru*-negative ORNs (Fig 4A). If this is the case, then *alh* should be expressed in *fru*-negative at4 ORNs and excluded from their *fru*-positive sibling ORNs at the onset of *or* expression. To test this, we used three GAL4 enhancer trap lines. Two of the lines have P-element insertions upstream of the first exon of short *alh* transcripts. The third line has an insertion within the first intron of the short isoform at a similar site to that of *alh* ^*j8c8*^ (S3 Fig). All insertions are located within the fifth intron of the long isoforms (S3 Fig).

Developmental analysis of the expression of these *alh* ^*GAL4*^-driven UAS-CD8GFP reporters showed a highly dynamic but reproducible expression pattern of *alh* during development (Fig 4B). In the antennal imaginal discs at the third larval instar, Alh is expressed in the central ring, which houses ORN precursors (S6 Fig). Later, 40–50 h after puparium formation (APF), *alh* is expressed in most ORNs (Fig 4B). At this time, *alh*-positive ORNs can be seen in sensilla clustered in the lateral regions of the antenna where the at4 and ac4 sensilla are located in adults, as well as neurons in the sacculus. The expression pattern becomes more restricted as pupal development proceeds and is almost absent in the adult.

Our observations suggest that Alh refines and coordinates *Or* and *fru* expression among the ORNs in at4 and ac4 sensilla. Developmental analysis presented later in this paper shows that *Or47b* expression begins at 40 h APF. Colabeling *alh* and *Or47b* showed that after 50 h, *alh* expression is excluded from *Or47b* ORNs while still present in other at4 ORNs (Fig 4C and 4D). The *alh* mutant phenotype can be observed at this same time, where clusters of *Or47b* and *fru*-positive 2–3 Or47b ORNs are observed in single sensilla (Fig 4E). In addition, the glomerular positioning defects of Or47b ORNs in the antennal lobe are apparent by 60 h APF (Fig 4F). These results suggest that *fruitless* expression is developmentally coregulated with *fru*-positive OR expression, and that Alh acts during development to repress the *fru*-positive OR identity in *fru*-negative ORNs prior to selection of their appropriate receptors.

### *Fruitless* Expression in ORNs Requires OR Function

In *alh* mutants, *fru* expression in the antenna is expanded alongside *Or47b* and *Ir84a* expression (Fig 3). The synchronous, expanded expression of both *fru* and *fru*-positive ORs in *alh* mutants suggests a tight relationship between OR and *fru* expression, regulated by Alh. We posited three possible ways to explain this relationship: 1) Alh represses *fru*, which is required to activate *Or* expression; 2) Alh represses *Or* expression, which can activate *fru* expression; and 3) Alh represses both *fru* and *Or* expression either directly or indirectly, by repressing an activator of both genes. Given the established role of Fru in the regulation of gene expression and courtship [33], our initial prediction was that Fru functions to regulate the expression of Or genes involved in courtship behaviors.

**Fig 5:**
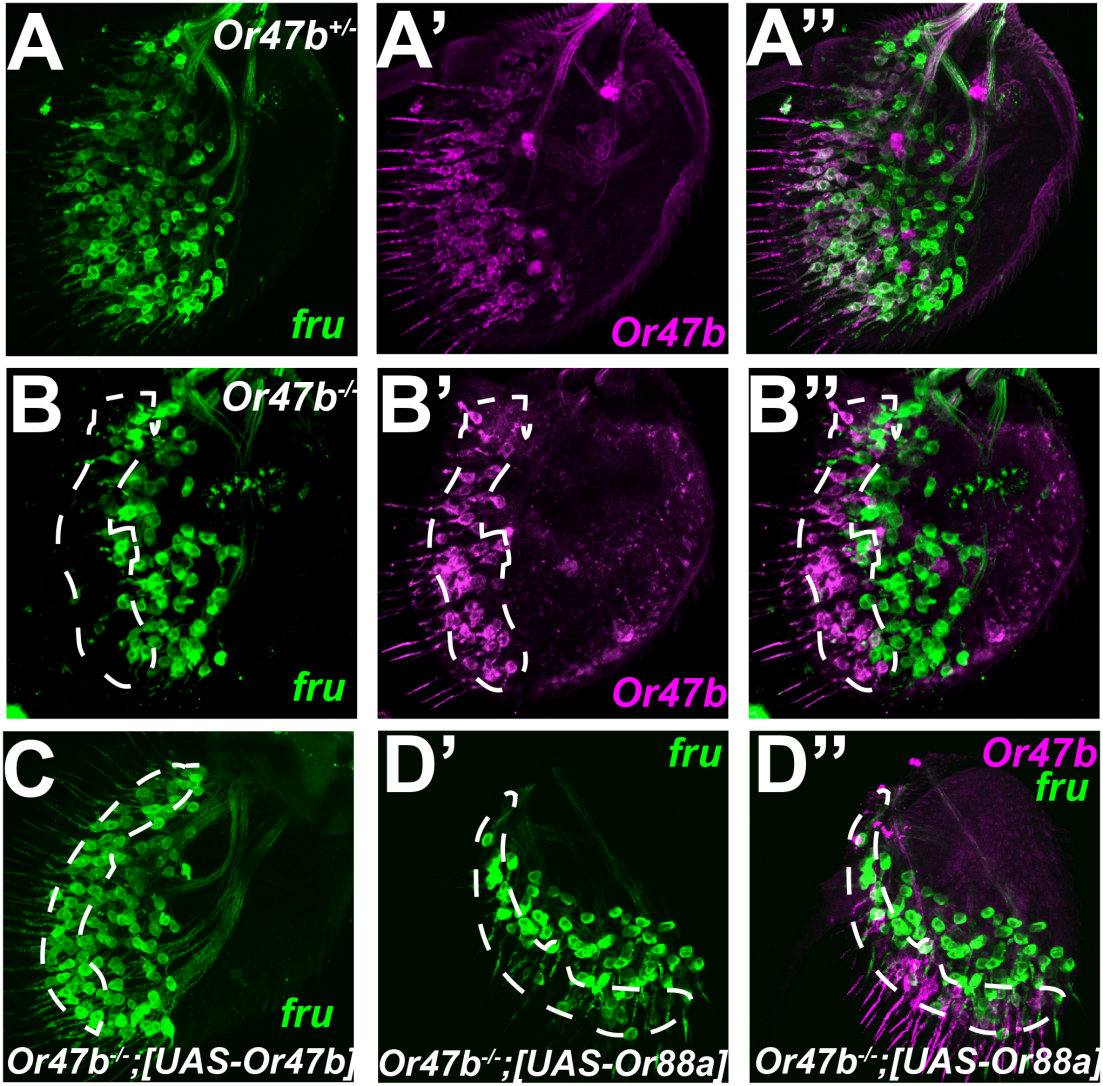
*fru* expression in adult Or47b ORNs requires Or47b function. **(A)** Heterozygous *Or47b* mutant antennae (3–5 d old) expressing *fruGal4 40XUASCD8GFP* **(A)** and *OR47b-CD2* **(A’)**. **(A”)** shows the merge of two images. **(B)** Homozygous *Or47b* mutant antennae (3–5 d old). **(C)** Overexpression of *UAS-Or47b* under the control of *fruGal4* in *Or47b* mutants (14 d old). **(D)** Overexpression of *UAS-Or88a* under the control of *fruGal4* in *Or47b* mutants (14 d old). GENOTYPES: A–A”: *Or47b-CD2 Or47b* ^*2*^ */+;fru* ^*GAL4*^ *UAS-40XCD8GFP/+* B–B”: *Or47b-CD2 Or47b* ^*2*^ */or47b* ^*2*^;*fru* ^*GAL4*^ *UAS-40XCD8GFP/+* C: *Or47b* ^*2*^ */Or47b* ^*2*^;*fru* ^*GAL4*^ *UAS-40XCD8GFP/UAS-Or47b* D: *Or47b-CD2 Or47b* ^*2*^ */Or47b* ^*2*^;*fru* ^*GAL4*^ *UAS-40XCD8GFP/UAS-Or88a*

To test this hypothesis, we first asked whether Fru regulates *Or47b* expression in Or47b ORNs. However, we found that *Or47b* expression is unaffected in *fru* mutants, suggesting that Fru does not regulate *Or47b* expression (S7 Fig). We then examined *fru* expression in the absence of Or47b function. Surprisingly, we observed that *fru* expression in Or47b ORNs is abolished in *Or47b* mutants, (Fig 5B). This finding was confirmed by qRT-PCR (S8 Fig). This phenotype could be rescued by the expression of a *UAS-Or47b* transgene driven with *fru* ^*GAL4*^ (Fig 5C). The expression of *UAS-Or88a*, a closely related receptor that detects similar ligands to Or47b [17] was partially able to rescue *fru* expression. This indicates a requirement for the function of ORs activated by the same ligand in *fru* regulation (Fig 5D and 5D’ and S11 Fig). Expression of *Or67d*, the third *fru*-expressing *Or* gene, which detects cVA, was not able to rescue *fru* expression in Or47b ORNs (S9D Fig).

**Fig 6:**
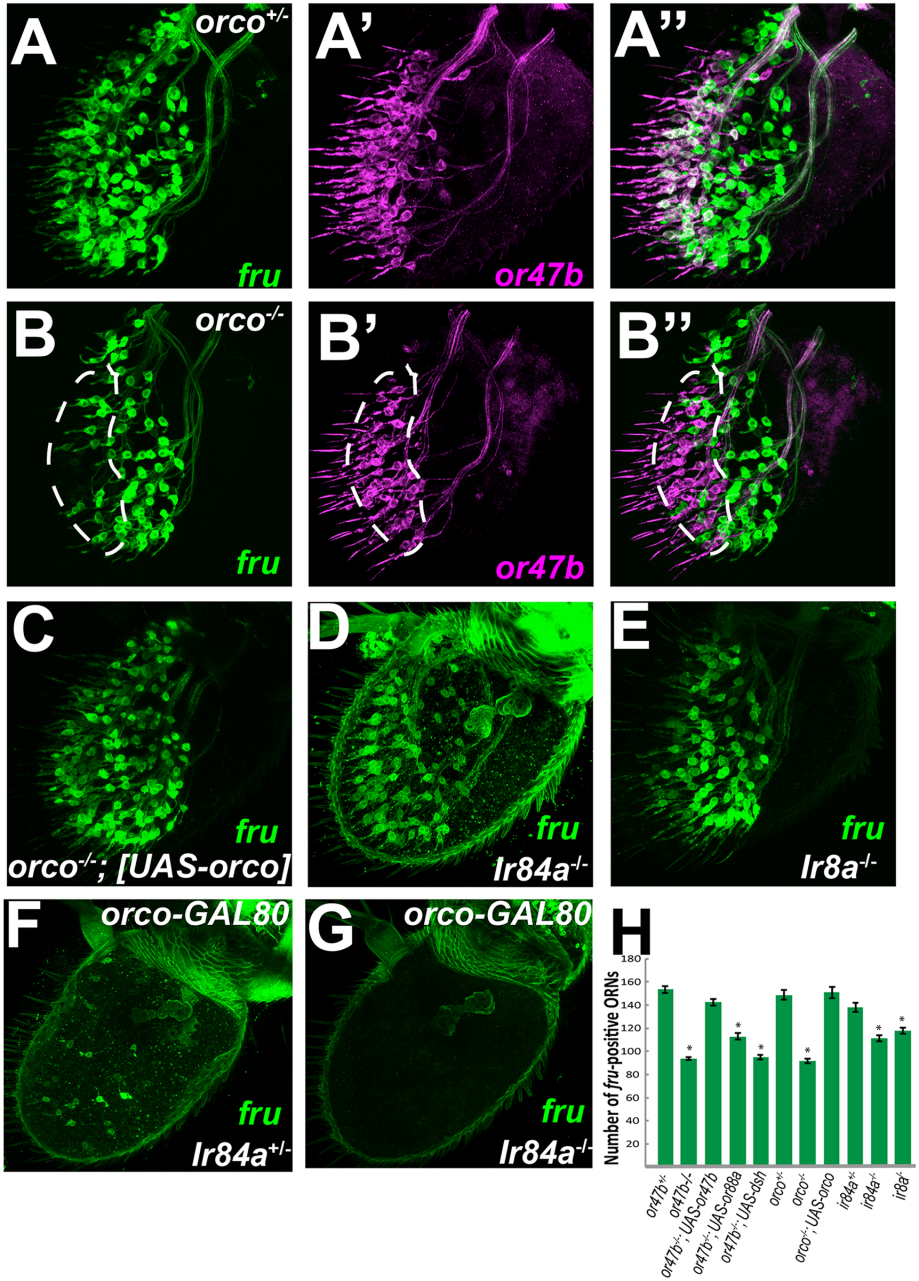
Or and Ir function is required to regulate *fru* expression in the adult olfactory system. **(A)** Heterozygous *orco* mutant antennae (3–5 d old) expressing *fruGal4 40XUASCD8GFP* **(A)** and *OR47b-CD2* **(A’)**. **(A”)** shows the merge of two images. **(B)** Homozygous *orco* mutant antennae (3–5 d old). **(C)** Overexpression of *UAS-orco* under the control of *fruGal4* in *orco* mutants (14 d old). **(D)** Homozygous *Ir84a* mutant antennae (3–5 d old) expressing *fruGal4 40XUASCD8GFP*. **(E)** Homozygous *Ir8a* mutant antennae (3–5 d old) expressing *fruGal4 40XUASCD8GFP*. **(F)** Heterozygous *Ir84a* mutant antennae (3–5 d old) expressing *fruGal4 40XUASCD8GFP and orco-Gal80*. **(G)** Heterozygous *Ir8a* mutant antennae (3–5 d old) expressing *fruGal4 40XUASCD8GFP and orco-Gal80*. **(H)** Quantification and statistical analysis of *fru*-positive ORN cell bodies observed in adult antennae of the indicated genotypes. *n* = 5–27. For all graphs, asterisks indicate significant (*p* < .005) differences from *or47b* heterozygotes. Error bars represent SEM. A one-way ANOVA was performed and followed with Tukey’s HSD—see Materials and Methods. All count data may be found in the Supporting Information as S1 Data. GENOTYPES: A–A”: *Or47b-CD2 /+;orco* ^*1*^ *fru* ^*GAL4*^ *UAS-40XCD8GFP /+* B–B”: *Or47b-CD2 /+;orco* ^*1*^ *fru* ^*GAL4*^ *UAS-40XCD8GFP /orco* ^*1*^ C: *+/ UAS-orco; orco* ^*1*^ *fru* ^*GAL4*^ *UAS-40XCD8GFP /orco* ^*1*^ D: *Ir84a* ^*MI00501*^ *fru* ^*GAL4*^ *UAS-40XCD8GFP / Ir84a* ^*MI00501*^ E: *Ir8a* ^*1*^ */Y; fru* ^*GAL4*^ *UAS-40XCD8GFP* F: *orco-GAL80/+; Ir84a* ^*MI00501*^ *fru* ^*GAL4*^ *UAS-40XCD8GFP / +* G: *orco-GAL80/+; Ir84a* ^*MI00501*^ *fru* ^*GAL4*^ *UAS-40XCD8GFP / Ir84a* ^*MI00501*^

We also abolished Or47b function using mutants for the general Or coreceptor *orco* [28]. A loss of *fru* expression, similar to that seen in *Or47b* mutants, was observed in *orco* mutants (Figs 6A, 6B and 6H, 8E and S11 Fig) and was confirmed by qRT-PCR (S8 Fig). This phenotype could be rescued by expression of a *UAS-orco* transgene (Figs 6C and 6H, 8E and S11 Fig).

Time-course analysis of antennae showed that *Or47b* mutant ORNs start showing signs of degeneration by 14 d (S9 Fig). A degeneration phenotype has been reported for *orco* mutants as well [34]. In order to show that loss of *fru* expression is not due to degeneration or neuronal death, we colabeled *Or47b* and *fru*–positive ORNs. Double-labeling experiments confirmed that in both *Or47b* and *orco* mutants, *fru* expression was dramatically decreased specifically in Or47b ORNs, and that this loss of *fru* occurred prior to neuronal degeneration (Figs 5B’, 6B’, 8E and S9, S11 Figs). Perturbing Wingless signaling has been shown to suppress *orco*-dependent neuronal degeneration [34]. *fru* ^*GAL4*^-dependent overexpression of *disheveled* (*dsh*), a downstream effector of the Wingless pathway, rescues the degeneration defect but not loss of *fru* expression in Or47b ORNs (S9C Fig). Expression of *Or88a* was sufficient to rescue Or47b ORN degeneration, and was also partially able to rescue loss of *fru* expression (Figs 5D, 8E, S9A and S9B and S11 Figs).

We next tested whether the other *fru*-positive receptors Ir84a and Or67d also regulate *fru* expression in adult ORNs. Unlike *Or47b* ORNs, *Or67d* and *orco* mutants did not affect *fru* expression in *Or67d* ORNs (S9D Fig). On the other hand, analysis of *fru* expression in *Ir84a* mutants and mutants of *Ir8a* (a coreceptor expressed in Ir84a ORNs [35]) showed that the number of *fru*-positive cell bodies decreases by approximately 20 cells (Fig 6D, 6E and 6H). To confirm that the decrease observed in *fru* expression in these mutants is restricted to Ir84a ORNs, we used *orco-GAL80* to suppress *fru* ^*GAL4*^-driven GFP expression in basiconic and trichoid ORNs. This allowed us to observe *fru* expression only in the Ir84a ORN population of wild type flies and *Ir84a* mutants. Wild type antennae had approximately 20 *fru*-positive cell bodies, which represent the Ir84a ORN class (Fig 6F and 6G). On the other hand, we observed a few to no *fru*-positive cell bodies in *Ir84a* mutants (Fig 6F and 6G). These findings were confirmed by qRT-PCR (S8 Fig). These results suggest that in addition to Or47b, Ir84a function is also required for *fru* expression in adult Ir84a ORNs.

### OR Function Is Required for Maintenance of *fru* Expression in Adult ORNs but Not during Development

**Fig 7:**
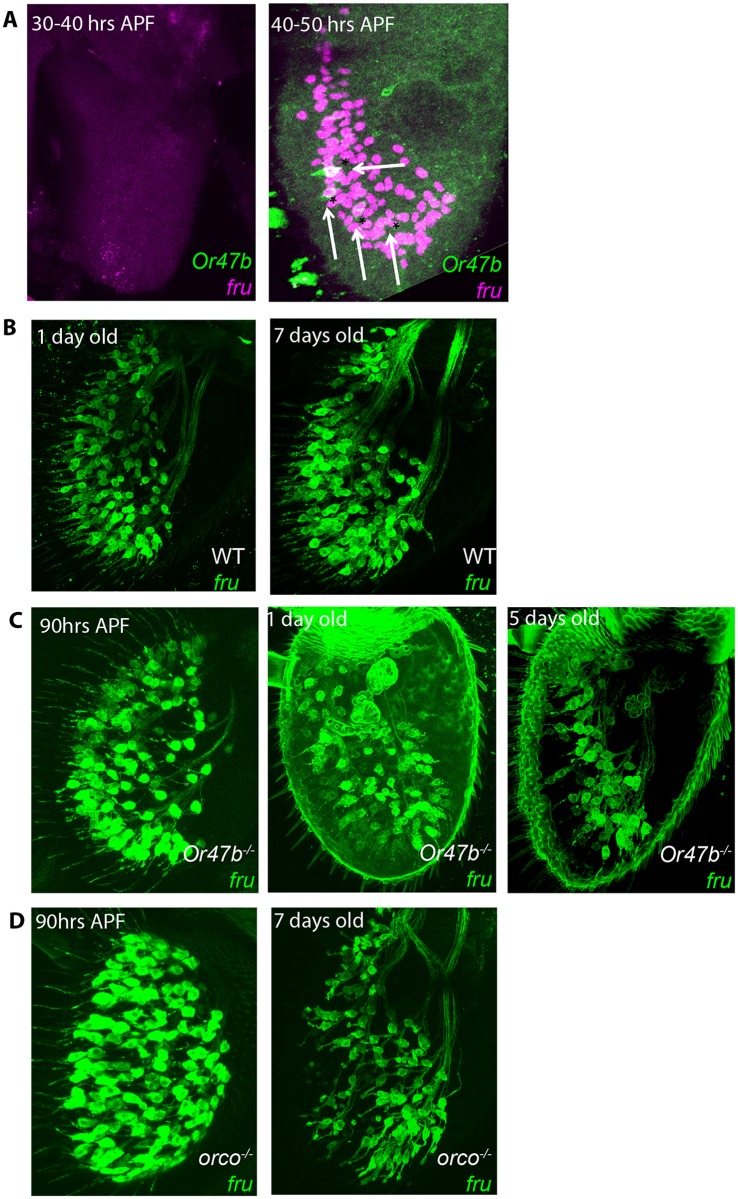
Onset of *fru* in developing ORNs overlaps with *Or47b* but is independent of *Or47b* function. **(A)** Wild-type antennae expressing *fruGal4 UAS-Redstinger* (magenta) and *Or47b-CD8GFP* (green). **(B)** Wild-type antennae expressing *fruGal4 40XUASCD8GFP*. **(C)** *Or47b* mutant antennae expressing *fruGal4 40XUASCD8GFP*. **(D)** *orco* mutant antennae expressing *fruGal4 40XUASCD8GFP*. GENOTYPES: (A) Or47b-CD8GFP/+; fru^GAL4^ UAS-RedStinger/+ (B) +/+; fru^GAL4^ UAS-40XCD8GFP (C) Or47b^2^/Or47b^2^; fru^GAL4^ UAS-40XCD8GFP (D) orco^2^/orco^2^; fru^GAL4^ UAS-40XCD8GFP

We predicted that both *fru* and *Or* expression must be established in pupal stages as ORNs adopt their final fates (Fig 4A). In wild type flies, the onset of *fru* expression coincides with the onset of *Or47b* expression, starting around 40 h APF and absent at earlier stages (Fig 7A). Given the temporal coordination of *Or47b* expression with *fru* during pupal development, we wanted to test whether Or47b function is required for the onset of *fru* expression. To do this, we analyzed *fru* expression during pupal development in *Or47b* and *orco* mutants (Fig 7). In these mutants, we found that *fru* expression in Or47b ORNs was still detectable by 90 h APF, suggesting that OR function is not required to initiate *fru* expression and that other factors, such as Alh, establish the correct expression and coupling of *fru/Or* during ORN development (Fig 7C and 7D). However, *fru* expression was lost within a few days after eclosion in both *Or47b* and *orco* mutants (Fig 7C and 7D). These results indicate that Or47b function is dispensable during the onset of *fru* expression in pupal stages, but once the flies eclose from their pupal cases, Or47b activity is required to maintain *fru* expression.

### *CamK1 and p300/CBP* Are Involved in Olfactory-Receptor-Dependent Maintenance *of fru* Expression

**Fig 8:**
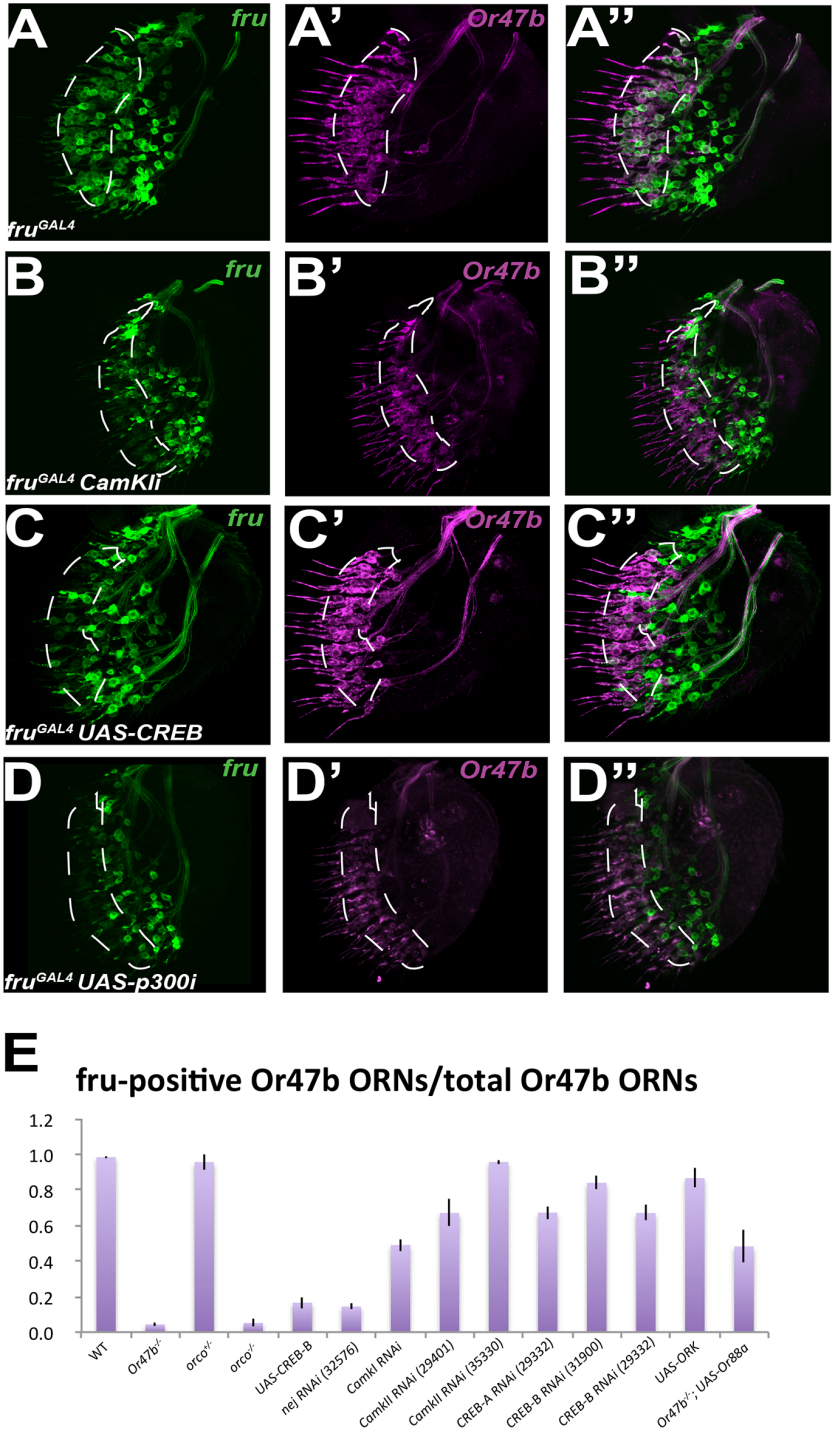
Maintenance of *fru* expression in adult ORNs requires CamK signaling and p300/CBP. **(A)** Wild-type antennae expressing *fruGal4 UAS-40XUASGFP* (green) and *Or47b-CD2* (magenta). **(B–D)** Antennae expressing *fruGal4 UAS-40XUASGFP* (green) and *Or47b-CD2* (magenta) as well as *fruGal4 CamKI* RNAi **(B)**, *UAS-creb* **(C)**, and *UAS-p300* RNAi **(D)**. **(E)** Quantification of antennal *fru*-positive ORN cell counts for experiments in Figs 5, 6 and 8. Data shown represents the fraction of *Or47b*-positive cells that are also *fru*-positive. For all graphs, asterisks indicate significant (*p* < .01) differences from *fru* ^*Gal4*^. Error bars represent SEM. A one-way ANOVA was performed and followed with Tukey’s HSD—see Materials and Methods. Cell count data also graphed in S10 Fig. All raw count data may be found in the Supporting Information as S1 Data. GENOTYPES: (A) *Or47b-CD2* /*+; fru* ^*GAL4*^ *UAS-40XCD8GFP* (B) UAS-CamKI RNAi/+; Or47b-CD2/+; fru^GAL4^ UAS-40XCD8GFP (C) UAS-CREB/+; Or47b-CD2/+; fru^GAL4^ UAS-40XCD8GFP (D) UAS-p300RNAi/+; Or47b-CD2/+; fru^GAL4^ UAS-40XCD8GFP

In *D*. *melanogaster*, ORs are necessary to generate both spontaneous and evoked patterns of ORN activity [9,13,28,36,37]. One possible explanation for the Or-dependent maintenance of *fru* expression in adult ORNs is that Or-mediated neuronal activity is required. To test the role of neuronal excitability in the regulation of *fru* expression, we electrically silenced *fru*-positive ORNs using the potassium channel *UAS-dORK*. We observed a statistically significant decrease in the number of *fru*-positive ORN cell bodies in the antenna when these ORNs were electrically silenced (S10A, S11 Figs and Fig 8E). However, double-labeling showed that this decrease was due to a smaller total number of Or47b ORNs. Unlike the OR mutants, the remaining Or47b ORNs continued to express *fru*. These results suggest that maintenance of *fru* expression in a subpopulation of ORNs requires OR function independent of neuronal activity.

Next, we investigated the mechanisms that relay Or/Ir activity to the transcriptional machinery in the nucleus. Unlike their mammalian counterparts, odorant receptors in *D*. *melanogaster* are not G-protein coupled but instead encode ligand-gated cation channels that conduct both calcium and sodium [38–41]. Given the role of calcium in signaling- and activity-dependent regulation of gene expression, we predicted that calcium signaling could maintain *fru* transcription in adult ORNs. To test this hypothesis, we screened RNAi knock-down lines of CamKI and CamKII using double-labeling of *Or47b* and *fru* expression. We found that disrupting CamKI function using different *UAS-CamkI RNAi* lines resulted in a decrease in *fru* expression in the Or47b ORN zone (Fig 8B and 8E, S11 Fig). Loss of CamKII had little yet significant effect on *fru* expression in Or47b ORNs as well (S10 Fig, S11 Fig). These results suggest that calcium signaling through CamKs contribute to maintenance of *fru* expression in a proportion of *fru*-positive ORNs.

CamKI encodes the *D*. *melanogaster* orthologue of the vertebrate CamKI/IV, which has been shown to phosphorylate the histone acetyl transferase CBP/p300 and CREB, both of which are activated by receptor signaling and can function to maintain gene expression [42–44]. We first tested the candidate gene *Creb-B*, which functions in signal-dependent regulation of gene expression in many contexts [45,46]. Overexpression of a dominant negative form of *Creb-B*, which was previously shown to abolish Creb function [47], did not result a change in *fru* expression (S10B Fig). RNAi-mediated knock down of either *Creb-A* or *Creb-B* had a mild yet significant decrease in *fru* expression in Or47b ORNs, similar to what was observed for CamKII RNAi knock down (Fig 8E, S11 Fig). In contrast, overexpression of *Creb-B* resulted in a substantial decrease in *fru* expression in Or47b ORNs (Fig 8C and 8E and S11 Fig).

**Fig 9:**
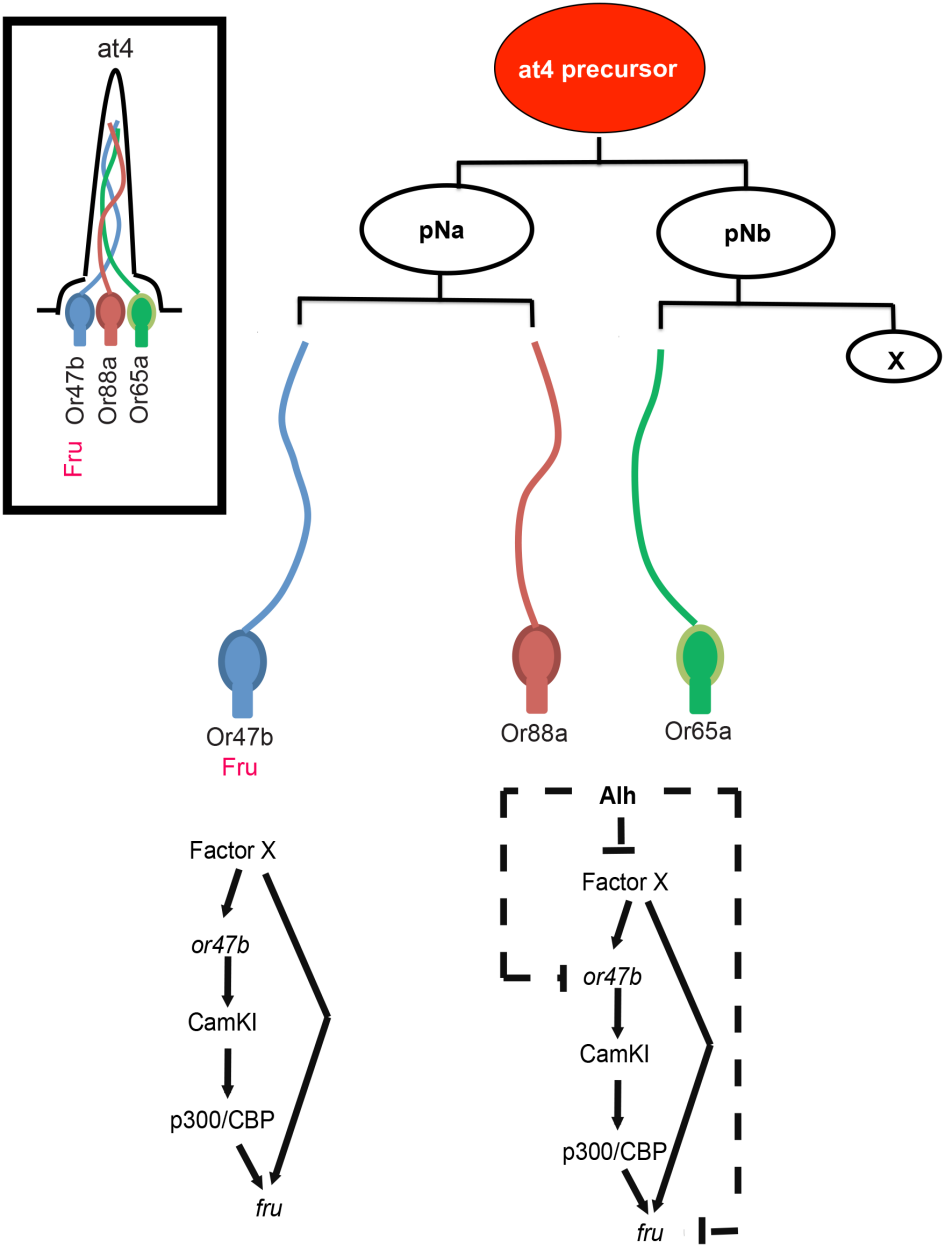
Regulatory feed forward loops establish and maintain *fru* expression in the olfactory system. A multipotent precursor cell divides asymmetrically to generate at4 ORN cell types. In Or47b ORNs, a factor X is required to coactivate both *Or47b* and *fru* expression during development. In Or65a and Or88a ORNs, Alh, either directly or indirectly through repression of X, is required to repress both *Or47b* and *fru*. Once *Or47b* and *fru* expression is established in Or47b ORNs, OR function maintains *fru* expression through *p300*/*CBP*.

The finding that *Creb-B* overexpression, but C*reb-B* mutants, shows a dramatic reduction of *fru* expression is contradictory to the known function of Creb-B as a transcriptional activator. These results suggested that the effect of Creb-B overexpression on *fru* regulation could be indirect. Thus, we tested the possibility that Creb-B might be titrating a *fru* regulator that is found in limiting amounts in the ORNs. Creb proteins are known to interact with CBP/p300, which also interacts with many other transcription factors to maintain gene expression [45]. In addition, titrations effects on gene expression due to competition for limiting amounts of CBP/p300 was previously reported [48–52]. Since CBP/p300 is activated through phosphorylation by CamKI [43], we tested the hypothesis that p300 is required to maintain *fru* expression in Or47b and Ir84a ORNs. Similar to the *CamKI* mutants, and C*reb-B* overexpression experiments, an RNAi line targeting p300 showed a strong decrease in *fru*-positive Or47b ORNs (Fig 8D and 8E, S11 Fig). These results suggest that OR as well as CamK signaling, and CBP/p300 histone acetyl transferase function contribute to the maintenance of *fru* expression in Or47b ORNs (Fig 9).

## Discussion

### *Fru* As a Molecular Identity Marker for Sex-Specific Behavioral Circuits

In *D*. *melanogaster*, sex-specific behaviors are largely regulated by a single gene, *fru*, whose expression is restricted to a circuit of approximately 2,000 interconnected neurons [3]. Functional studies of *fru*-positive neurons support the hypothesis that *fru* labels the neuronal circuits that drive sex-specific behaviors, but the mechanisms that restrict its expression in the nervous system are not known. If *fru* is necessary for appropriate circuit formation during development as well as circuit function, its expression in appropriate neurons must be tightly controlled using hardwired developmental programs for neuronal fate such as OR expression. However, it is also known that sex-specific behaviors are adaptable, suggesting a need for a plastic molecular mechanism to modify *fru* expression and the structure of *fru*-positive circuits. In this paper, we describe two different modes of transcriptional regulation of *fru* in ORNs mediating sex-specific behaviors: (1) establishment of *fru* expression through an Alh-mediated chromatin modulatory pathway that coordinates and refines *fru*, *Or47b*, and *Ir84a* expression in developing ORNs; and (2) maintenance of *fru* expression through ORs in adult ORNs by calcium signaling and histone acetyl transferase, p300/CBP. To our knowledge, our results provide the first example of a regulatory pathway that coordinates the sensory identity of neurons with the identity of the neural circuit they must integrate into. Our results suggest that this is a distinct step in the developmental program of a neural circuit, one that is independent of axon guidance decisions by the neurons as the circuit develops. These findings also highlight the role of feed-forward regulatory loops that establish and maintain these identities with hardwired and environmentally sensitive components, respectively (Fig 9).

### Separate Programs Control Odorant Receptor Choice and Axonal Guidance during ORN development

In mammals, OR genes are critical for appropriate axon guidance through an elegant mechanism that links the amount of guidance molecules produced by the axon to the unique, ligand-independent G-protein coupled receptor (GPCR) activity signature of each Or [53–55]. These guidance molecules interact with gradients of other guidance molecules to sort ORN axons in a class-specific manner as they enter the olfactory bulb [53–55]. In contrast, OR function does not regulate ORN targeting in *D*. *melanogaster*. Many groups have shown that there is no contribution of Or genes to the guidance of ORN axons to specific glomerular zones in *D*. *melanogaster* [9,27,28,36,37]. The fact that axon guidance is largely completed before OR expression begins [29] also supports the hypothesis that programs of sensory receptor choice and axon guidance are independently regulated.

Nevertheless, receptor identity must be coupled with an appropriate target location to establish the one-neuron/one-receptor/one-glomerulus rule for odortopic mapping in the brain. Since the identity of the ORN precursor cell determines both the receptor expression and guidance programs for all the ORNs it will generate, the expression and axon guidance instructions for each ORN must be segregated together during asymmetric divisions of these precursors. Notch signaling, which acts in a context-independent manner during these divisions, is required to ensure segregation of both sensory and glomerular targeting identities of ORNs [20,22]. The sorting of *Or* expression and guidance fates throughout precursor division is accomplished through the generation of Notch “ON” and “OFF” cellular states due to asymmetries in Notch signaling components [20,56]. Perturbing Notch function results in the duplication of both sensory fates and glomerular postion of one ORN class at the expense of the other within a sensillum [20]. Segregation of glomerular positioning programs for ORNs in the same sensillum during asymmetric precursor divisions requires Notch-dependent differences in the repulsive cell surface molecule *sema-2b* [57]. Notch was also shown to affect postmitotic neuronal targeting in the *D*. *melanogaster* central nervous system independent of neuronal fate decisions [58]. However, very little is known about the mechanisms that segregate sensory identity of ORNs downstream of Notch. *alhambra* is the first gene identified that, when mutated, results in a complete conversion of the sensory identity of one ORN in the same sensillum to another while remaining independent of glomerular positioning decisions. This indicates that Alh specifically regulates the proper segregation of individual *Or* choice programs among ORNs in the same sensillum after glomerular positioning decisions are properly segregated and established. It will be interesting to see whether *Or* and *fru* expression decisions driven by Alh are induced downstream of Notch signaling.

### The Putative Chromatin Modulator *alhambra* Coregulates *OR* and *fru* Expression

Alh encodes the *D*. *melanogaster* orthologue of the leukemia fusion gene AF10, which has been previously shown to interact with chromatin modulators such as proteins in the SWI/SNF remodeling complex and the histone methyl transferase hDOT1 [59,60]. More recently, it was shown that the *Caenorhabditis elegans* orthologue of *alhambra*, ZFP-1, interacts with Dot1 and affects cell signaling by regulating RNA polymerase pausing on selected genes [61]. Recent work suggests that polymerase pausing may have many effects on transcriptional regulation, and one such effect appears to be an establishment of permissive chromatin around the paused polymerase [62].

A key feature of the *alh* mutant phenotype is the repressive effect it has on the expression pattern of both *fru* and *fru*-positive OR genes during development. The loss of ORN diversity within the at4 and ac4 sensilla suggests that Alh might modify chromatin in response to signals such as Notch, which operate during asymmetric cell divisions to diversify ORN fates. The phenotypic specificity of *alh* mutants to at4 and ac4 *fru*-positive sensilla suggests that Alh functions during the development of these sensilla to ensure *fru* expression is restricted to only certain neurons that are tuned to specific odors. Alh may function to repress the expression of terminal differentiation genes that work together to define the odor tuning properties of a given ORN (Ors) and the behavioral circuits it will be integrated into (Fru), independently of axon guidance programs (Fig 9).

It is likely that a factor X (Fig 9) is expressed in Or47b ORNs that coordinately activates *fru* and *Or47b*. The restriction of the sensory conversion phenotype in *alh* mutants to *fru*-positive ORs suggests that Alh might suppress the *fru*-positive ORs (e.g., Or47b) and promote alternate OR expression (e.g., *Or88a*), either indirectly, through suppression of factor X, or directly prior to the selection of an alternate receptor by the ORNs in the same sensillum (Fig 9). For example, the *fru*-positive Or47b identity might be the “default,” since the expression of either *Or65a* or *Or88a* seems to require the suppression of *Or47b*, and *Or47b* is the first receptor to be expressed in at4 ORNs. Once Or47b expression begins in one of the at4 ORNs, Notch signaling may ensure that *Or47b* is turned off in the sibling ORNs or precursors by changes in chromatin induced by proteins such as Alh. It is interesting to note that a “default” identity from which other identities diversify is also a phenomenon seen in the diversification of ORN precursor identities, pointing to a modularity in the cell fate programs regulating ORN diversity [18]. The phenotype is slightly more complex for *Ir84a*, where *fru*-positive *Ir84a* expression not only expands to another ORN in the same sensilla subtype but also to an ORN in another coeloconic sensilla. The reason for this discrepancy might be due the differences in developmental programs of coeloconic sensilla ORNs expressing Irs. Several *Irs* are in fact expressed in multiple neurons across several different coeloconic sensilla subtypes, a phenomenon not seen in canonical ORs expression [32,40]. The third *fru*-positive OR, *Or67d*, is not affected in *alh* mutants. This might be due to the fact that this is the only ORN in the at1 sensillum.

The expansion of OR expression to other sensilla ORNs without affecting guidance was previously reported in *atrophin* mutants [63]. However, there are multiple differences between the *atrophin* and *alh* mutant phenotypes. First, in *alh* mutants, in both ac4 and at4 sensilla, there is a within-sensilla sensory identity conversion among developmentally related ORNs. In *atrophin* mutants, expansion does not happen among the ORNs within the same sensillum. Secondly, in *atrophin* mutants, there is a coexpression of both the endogenous receptor and the ectopically expressed receptor in the affected ORNs. In contrast, in *alh* mutants, the expansion of one receptor to ectopic ORNs is accompanied by the loss of the endogenous receptor normally expressed in that ORN. Thus, the mutant ORNs express a single receptor rather than two. Loss of *alh* function converts the sensory identity to one at the expense of another, whereas in *atrophin* mutants, ORNs maintain a dual sensory identity.

### OR Signaling in the Maintenance of *fru* Expression in Adult Flies

Here, we report for the first time that once the terminal differentiation of Or47b and Ir84a ORNs is complete, OR signaling is required for the maintenance of *fru* expression. *fru* expression is lost in an ORN class-specific manner in *Or47b* and *Ir84a* mutant adults. This process also requires CamK signaling and the histone acetyl transferase *CBP/p300*, as mutants in both are associated with a loss of *fru* expression in adult ORNs. Interestingly, *fru* expression does not require OR function in Or67d ORNs. This raises the possibility that *fru* expression in Or67d ORNs is somehow hardwired and not under the control of OR activity.

What, then, is special about Or47b and Ir84a ORNs? *fru* expression in Or67d ORNs appears unaffected in *orco* and *Or67d* mutants. Given the well-established role of Or67d in detection of the male specific pheromone cVA and regulation of courtship, it is possible that *fru* expression is under a different and more robust regulatory mechanism in these ORNs, one that is independent of OR signaling. Previous studies on the patterns of projection neurons (PNs), which synapse with the *fru*-positive ORNs have also shown that the organization of Or67d circuit is somehow different from Or47b and Ir84a circuits deeper in the brain. In the lateral horn, a downstream processing center, the axons of the PNs that synapse with Or47b and Ir84a ORNs closely overlap. In fact, they overlap more than any of the other classes of PNs getting input from the 44 ORN classes, while remaining excluded from the Or67d PNs terminals [64]. Aside from these anatomical differences which may indicate possible differences in function, ligand activation of Or and calcium signaling may underlie the Or47b- and Ir84a-dependent maintenance of *fru* expression. In *orco* and *Or47b* mutants, *fru* expression is lost soon after flies eclose from the pupal case, which might indicate odor-dependent activation of ORs in this process.

Our findings that the expression of *Or88a*, but not *Or67d*, in *Or47b* mutant ORNs can partially rescue the loss of *fru* expression in *Or47b* mutants also support the idea Ors are not entirely interchangeable, and that detection of specific fly odors function to maintain *fru* expression. Recent work has at long last identified the ligands that activate Or47b and Or88a ORNs [17]. While both ORNs have long been known to respond to the complex mixture of compounds found on both male and female cuticles [64], this work establishes that both Or47b and Or88a ORNs respond robustly to a single compound, methyl laurate. On the other hand, Or67d ORNs are activated by cVA, but not by methyl laurate. These responses suggest that the similarity of the ligands detected by Or47b and Or88a ORNs may underlie Or88a’s ability to partially rescue the maintenance of *fru* expression.

Despite the innate aspects of courtship behaviors and the role of Fru^M^ in regulating structure and function of courtship circuits, there is an adaptable aspect to courtship. A particularly interesting example of adaptive courtship behavior is a recently described type of experience-dependent courtship [65]. Males lacking Fru^M^ function that have been housed in isolation display very little courtship towards female or male targets. However, when *fru* mutant males are housed in groups, they acquire the ability to court a wide variety of courtship objects. This learned form of courtship appears to require olfactory input, as no experience-dependent courtship is observed in *fru-orco* double mutants. It is intriguing to speculate that Or47b, through detection of methyl laurate, may contribute to olfactory experience-dependent modulation of courtship behavior. The receptor-dependent plasticity of *fru* expression we describe in these ORNs may also be connected to the experience-dependent plasticity of courtship behavior. Recent identification of *fru*-dependent transcriptional targets showed that *fru* regulates the expression of many genes that affect different processes in neuronal development in addition to neuronal function [33]. The interconnectedness of *fru*-positive neuronal circuits [8] and the behavioral function of *fru* raises the possibility that *fru* might regulate the flow of information from one *fru*-positive neuron to another within the circuitry. Thus, loss of *fru* expression in *Or47b* or *Ir84a* mutants might interfere with the integration and communication of Or47b and Ir84a ORNs with the command centers regulating courtship behaviors.

### Structural Evolution of *ORN* Circuits Underlying Sex-Specific Behaviors

Finally, it is tempting to speculate that the processes we describe may operate across Drosophilid and even insect species. Or47b gene sequence and the ligands that activate Or47b and Or88a ORNs are well preserved across *Drosophila* species [17]. This might also explain the olfactory-experience-dependent courtship learning that occurs in *fru* ^*M*^ mutants and allows courtship with species other than *D*. *melanogaster* [65]. It is plausible to speculate that *fru* ^*M*^ expression is less robust in ORNs, which have more modulatory effects on courtship, like Or47b and Ir84a. In more innate pathways, driven by the Or67d-dependent detection of cVA, *fru* expression is independent of OR activity, more robust, and only regulated during development.

It is also important to note the anatomical similarities between the enlarged, *fru*-positive, dorsolateral portion of the antennal lobe in *alh* mutants and the macroglomerular complex previously described in *Manduca* antennal lobes [66]. This enlarged region of the moth antennal lobes is also located in the dorsolateral region and is the region responsive to bombykal, the female sex pheromone [66]. It is possible that in *alh* mutants, this structure is converted to a more ancient configuration similar to a macroglomerular complex, and that addition of Alh to the gene regulatory networks required for the assembly of the *D*. *melanogaster* ORN circuits to further diversify ORN fates from an ancestral state. The anatomical similarities across insect species as well as the conservation of fly-produced mating signals, invite speculation that odor-evoked plasticity is generated by the same mechanisms across insect species, and future studies are needed to investigate whether these mechanisms underlie plasticity of courtship behaviors.

## Materials and Methods

### Fly Genetics

Fly stocks were maintained on conventional cornmeal-agar-molasses medium at 25°C. The rationale for the genetic screen, from which the *alh* ^*1353*^ mutant was recovered, is described in [24]. *Or47b* mutants, UAS-*Or67d*, and UAS-*Or88a* were kindly provided by Leslie Vosshall, Dean Smith, and John Carlson, respectively. *fru* ^*GAL4*^ alleles were obtained from Barry Dickson and Bruce Baker. Other mutant alleles and transgenic lines used in MARCM analyses were obtained from Bloomington and Kyoto Stock Centers. *Or47b-LexA* transgene was generated by cloning in a 3-kb fragment of *Or47b* upstream regulatory regions into a LexA plasmid and were injected to obtain transgenics. The expression pattern of the *Or47b-LexA* transgene was confirmed to match the *Or47b-GAL4* line.

### Mapping *p1353* Mutation

The *p* ^*1353*^ mutation is homozygous lethal. Complementation tests with the deficiency kit on the right arm of third chromosome yielded four deficiencies (*Df(3R)Antp17*, *Df(3R)Antp6*, *Df (3R)Antp1* and *Df(3R)Scx4*) that failed to complement the lethality of the *p* ^*1353*^ allele, narrowing the cytological position to a region between 84B–D. Further complementation tests using single gene mutants in the region identified *alh* ^*j8c8*^ as allelic to the *p* ^*1353*^ mutation. The *alh* ^*j8c8*^ allele is a LacZ enhancer trap line inserted in the first intron of the second transcriptional start site of *alh*. Phenotypic characterization of *Or47b* expression in *alh* ^*j8c8*^ MARCM clones [30] showed that *alh* ^*j8c8*^ phenocopies the *p* ^*1353*^ mutation (Figs 1 and 2). The results from the complementation tests and phenotypic analysis suggest that *alh (AF10)* is the gene disrupted in *p* ^*1353*^ mutants. *alh* encodes the *D*. *melanogaster* orthologue of the leukemia fusion gene *AF10*, an epigenetic factor involved in heterochromatin-mediated gene silencing [67–69]. It has two major transcriptional start sites and multiple splice isoforms, which are either “long” isoforms containing the PHD domain, or short isoforms lacking this domain (S3 Fig). We refer to the *p* ^*1353*^ allele as *alh* ^*1353*^ in the paper.

### Immunohistochemistry

Staining of adult and pupal brains was performed as previously described [18,24]. Primary antibodies were used in the following dilutions: rabbit α-GFP 1:1,000 (Invitrogen), rat α-Ncad 1:20, rat α-elav 1:100, mouse α-Bruchpilot 1:20 (Developmental Studies Hybridoma Bank), mouse α-CD2 1:1,000 (Serotec). The following secondary antibodies were used: goat α-rabbit-FITC 1:1000, goat α-mouse-Cy3 1:100, goat α-rat-Cy3 1:200, goat α-guinea pig-Cy3 1:500, goat α-rat-Cy5 1:200, goat α-rabbit-Cy5 1:500 (Jackson ImmunoResearch), Alexa 568 goat α-mouse IgG highly cross-adsorbed 1:300 (Invitrogen). Confocal images were taken using an Olympus Fluoview FV1000.

### Statistical Analysis

Data was analyzed using JMP.

#### Total cell counts (Figs 1D–1F and 2B and 2F)

Cells were counted by an experimenter blinded to all genotypes.

A one-way ANOVA was performed for each genotype. Statistical significance was accepted at the *p* < .01 level.

*or47b*, F(2,89) = 79.7037, *p* < .0001.

*or88a*, F(2,131) = 23.8642, *p* < .0001.

*or65a*, F(2,49) = 15.1466, *p* < .0001.

*ir84a*, F(2,95) = 31.0864, *p* < .0001.

*or67d*, F(1,51) = 0.0786, *p* = .7804

All simple pairwise comparisons were made using the Tukey’s HSD test. Statistical significance was accepted at the *p* < .005 level for pairwise comparisons.

#### Cell cluster ratios (Fig 1G)

Cells were counted by an experimenter blinded to all genotypes.

A one-way ANOVA was performed. Statistical significance was accepted at the *p* < .01 level.

*or47b*, F(2,85) = 716.4228, *p* < .0001.

All simple pairwise comparisons were made using the Tukey’s HSD test. Statistical significance was accepted at the *p* < .005 level for pairwise comparisons.

#### *Fru*-positive cell counts (Fig 6H)

A one-way ANOVA was performed with all *fru* data: F(10,145) = 68.5683, *p* < .0001, followed by Tukey-Kramer HSD posthoc tests. Statistical significance was accepted at the *p* < .005 level for pairwise comparisons.

#### *Fru*-positive cell counts (Fig 8D)

A one-way ANOVA was performed with all *fru* data: *F*(13,172) = 43.3096, *p* < .0001, followed by Tukey-Kramer HSD posthoc tests. Statistical significance was accepted at the *p* < .005 level for pairwise comparisons.

### RT-PCR

Antennae from approximately 25 flies were dissected for each genotype, and at least two biological replicates were analyzed for each genotype. RNA was extracted with an RNeasy kit (Qiagen), treated with on-column DNase digestion (Qiagen), and then reverse transcribed into cDNA using the SuperScript First-Strand Synthesis System for RT-PCR (Invitrogen). qPCR was performed using the FastStart Universal SYBR Green Master Mix (Roche) using standard protocol. Expression for each gene was analyzed in triplicate using primers below (Table 1). Ct values were normalized to the expression of actin for each genotype, and these normalized values for experimental and control genotypes were then compared using the delta-delta Ct method. Significance was determined using paired, two-tailed *t* tests comparing Wt and mutant delta Ct values.

**Table 1:** qPCR primers.

Primer Name	Sequence
OR47b-qPCR-F	CAAATCTCAGCCTTCTGCGG
OR47b-qPCR-R	GATACTGGCACAGCAAACTCA
IR84a-qPCR-F	CAGTTGGTCAGGTGTGATGG
IR84a-qPCR-R	AAAGTGGATGTTCTGGGTGTG
OR65a-qPCR-F	TTGGGATCGATTGTTTGGACC
OR65a-qPCR-R	AACCTAGGGCTTTCAACTGGT
OR88a-qPCR-F	GGCGGTACCGGAAGTTCTAT
OR88a-qPCR-R	GCTGCATTATTTCAGTGAAGTGC
fruM-qPCR-F	CCCGCATCCCCTAGGTACAA
fruM-qPCR-R	GACTGTTTCGCCCTCGCAGG
fruC-qPCR-F	CAAATTTGACCGGCGTGCTAACCT
fruC-qPCR-R	AGTCGGAGCGGTAGTTCAGATTGT
orco-qPCR-F	GCCTAGATGATTGCTGCATTACT
orco-qPCR-R	CGAGGTTGTCATCCTTGCTATT
ACT5C-qPCR-F	GGCGCAGAGCAAGCGTGGTA
ACT5C-qPCR-R	GGGTGCCACACGCAGCTCAT

### Generating *Or67d* Mutants Using CRISPR

A targeted deletion of the Or67d coding region was generated using the CRISPR Cas9 system. Guide RNA sequences that flank the Or67d coding region were:

Or67d 5’ chiRNA sense oligo- 5’ CTTCGTGCTTTCGATTATTTTTCC 3’

Or67d 5’ chiRNA antisense oligo- 5’ AAACGGAAAAATAATCGAAAGCAC 3’

Or67d 3’ chiRNA sense oligo- 5’ CTTCGAAGGCCAAGATGGTTGCTG 3’

Or67d 3’ chiRNA antisense oligo- 5’ AAACCAGCAACCATCTTGGCCTTC 3’

The sense and antisense oligo for each chiRNA were annealed and then cloned into the BbsI site of the pU6-BbsI-chiRNA plasmid (Addgene). The Or67d locus was replaced with a 3xP3-DsRed selectable marker and attP phage recombination site by homology-directed repair. Homology arms of approximately 1 kb upstream and downstream of the Cas9 target sites were amplified from genomic DNA from the genotype into which they would be injected, y1 M vas-Cas9.GFP-ZH-2A w1118, using the following primers:

5’ homology arm forward- 5’ TTCCCACCTGCAAATTCGCTTACCCAAAAAGGGCGGCTG 3’

5’ homology arm reverse- 5’ CACACACCTGCCCCCCTACAAAATAATCGAAAGCGCCAC 3’

3’ homology arm forward- 5’ AGGCCTCTGAGGGGTGTTGGGAGGTC 3’

3’ homology arm reverse- 5’ GTGATTCTGCAGCTGCCAACGGGAAGCAATCT 3’

The homology arms were cloned into the 5’ and 3’ multiple cloning sites of the pHD-DsRed-attP plasmid (Addgene) with AarI (5’ arm), StuI (3’ arm) and PstI (3’ arm).

The embryo injection mixture contained the two pU6-BbsI-chiRNA plasmids at 100 ng/mL each and the pHD-DsRed-attP donor plasmid at 500 ng/mL. F1 progeny of the injected embryos were screened for DsRed expression in the adult eye and correct targeting of the Or67d locus was confirmed by PCR and DNA sequencing of the locus.

## Supporting Information

S1 Data Supporting data underlying graphs throughout the paper. (XLSX)

S1 Fig qPCR validation of *alh* ^*1353*^ phenotype. Data shown represents the fold change (normalized by the ΔΔC_t_ method) in the expression of selected genes in the antenna as compared to MARCM control flies. A value of 1 indicates no change from control. All fold change data may be found in the Supporting Information as S1 Data.
(TIF)

S2 Fig Glomerular innervation of ORN classes in *alh* ^*1353*^ mutants. Connectivity of ORNs in wild type (top panels) and *alh* ^*1353*^ mutant (bottom panels) antennal lobes.
(TIF)

S3 Fig *Alh* gene and protein structure. (A) *alh* splice isoforms (letters on the right denote the name of the isoform). The *Alh* ^*j8c8*^ allele and the *alh* ^*NP*^ lines used in expression analysis are inserted in the first intron of the short isoforms, and the fifth intron of the long isoforms. (B) Major protein domains found in long and short Alh isoforms.
(TIF)

S4 Fig Or88a cells in *alh* mutants do not express *fru*. A) Double-labeling of *Or47b* and *Or88a* in WT and *alh* mutant antennae and antennal lobes. *Or88a* expression is not expanded to other ORNs in *alh* mutant antennae. B) Double-labeling of *Or88a* and *fru* in WT and *alh* mutant antennae and antennal lobes. *Or88a* expression does not overlap with *fru* expression in wild type or *alh* mutant antennae. C) *Ir76a* expression is decreased in *alh* mutant antennae and antennal lobes.
(TIF)

S5 Fig Overexpression of alh isoforms does not affect expression of Or88a or Or47b. A) Expression of the long isoform of *alh* (*alh-L)* under the control of *elav-GAL4* does not change the expression of either *Or47b* and or *Or88a* as assayed with direct fusion reporters in adult male antennae. (Expression of the short isoform of *alh* (*alh-S)* under the control of *elav-GAL4* is lethal). B) Overexpression of the short isoform does not affect Or47b or Or88a expression. Flies were heat-shocked at 37°C for one hour during larval and pupal development at the indicated ages.
(TIF)

S6 Fig *Alh* expression in the developing larval eye-antennal disc. AlhGal4 expression in 3L larvae shows a spatial restriction to the center of the eye-antennal disc.
(TIF)

S7 Fig *Or47b* expression is unaffected in *fru* mutants. (TIF)

S8 Fig qPCR validation of *fru* expression in *Or47b* and related mutants. Data shown represents the fold change (normalized by the ΔΔC_t_ method) in the expression of selected genes in the antenna as compared to w^1118^ control flies. A value of 1 indicates no change from control. Asterisks indicate *p* < .05 as measured by two-tailed *t* tests comparing C_t_ values of each genotype to controls. All fold change data may be found in the Supporting Information as S1 Data.
(TIF)

S9 Fig ORN degeneration defects, and their rescue, observed in *Or47b* and *Ir84a* mutant 14-d-old antennae. (A) Degeneration defects in *Or47b* mutants are rescued by *UAS-dsh* and *UAS-Or88a* overexpression using *fru* ^*GAL4*^. (B) Expression of *Or88a* in *Or47b* mutants (bottom) partially rescues the loss of *fru*-positive ORN axon terminals (*fru-syTGFP)* in Or47b target glomerulus (top). (C) Loss of *fru* expression in *or47b* mutants is independent of neuronal degeneration. Degenerating Or47b ORN cell bodies are apparent by 14 d (left), which are rescued by overexpression of *UAS-dsh* (middle). However, despite the rescue of neuronal death, *fru* is still not expressed in *or47b* mutant antennae (right). (D) *Or67d* expression is not able to rescue the degeneration and *fru* expression defects in *Or47b* mutants (left panels). *Or67d* mutants do not have defects in *fru* expression and they do not degenerate by 14 d (right panels).
(TIF)

S10 Fig Neuronal activity, *CamKII* and *Creb* are not involved in maintaining *fru* expression. *Fru* expression is not affected by neuronal silencing using *fru* ^*GAL4*^ driven UAS-ORK expression (A), or loss of Creb2a (B) and CamkII (C) function using *fru* ^*GAL4*^-driven UAS-RNAi expression. Inhibition of *PKA* (D) and *Creb* (F) function by *fru* ^*GAL4*^-driven UAS-*PKA* ^*inhibitor*^ and UAS-*CREB* ^*DN*^ expression, respectively, also does not affect *fru* expression. Constitutive activation of *CamKII* also does not have an effect (E).
(TIF)

S11 Fig Distribution of *fru*-positive and *fru*-negative Or47b ORNs in different mutant backgrounds. See Fig 8 in main text for significance. All raw count data may be found in the Supporting Information as S1 Data.
(TIF)

## Abbreviations

APF: after pupanium formation
CBP: CREB Binding Protein
cVA: cis-vaccenyl acetate
GPCR: G-protein coupled receptor
HS: Honest Significant Difference
OR: olfactory receptor
ORN: olfactory receptor neuron
PN: projection neuron
SEM: standard error of the mean
