# Supplementary figures and images for: Chromatin Modulatory Proteins and Olfactory Receptor Signaling in the Refinement and Maintenance of Fruitless Expression in Olfactory Receptor Neurons
Source: PLoS Biol. 2016 Apr 19;14(4):e1002443. doi: 10.1371/journal.pbio.1002443 (PMC4836687; doi:10.1371/journal.pbio.1002443)

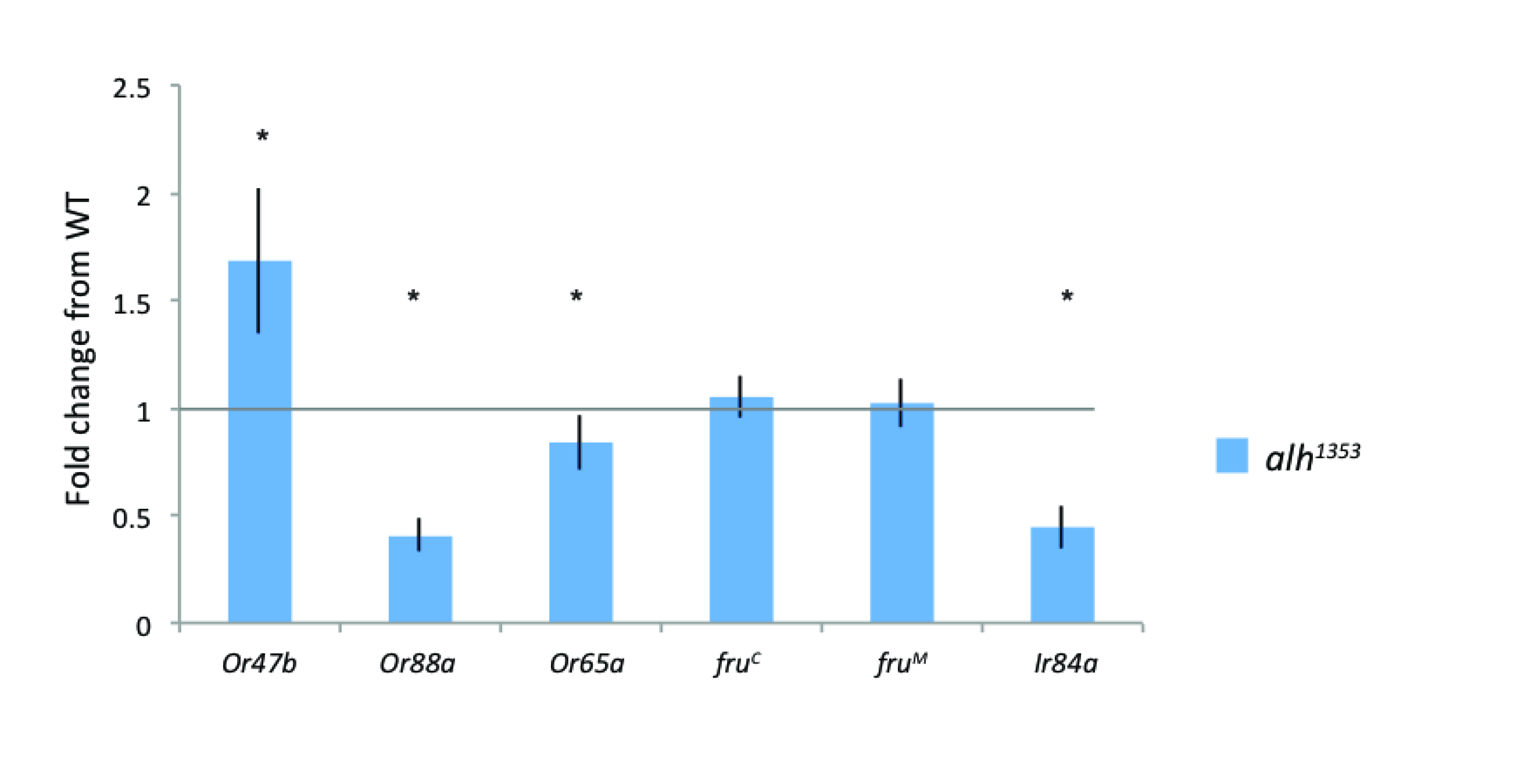

Supplement: S1 Fig — Data shown represents the fold change (normalized by the ΔΔCt method) in the expression of selected genes in the antenna as compared to MARCM control flies. A value of 1 indicates no change from control. All fold change data may be found in the Supporting Information as S1 Data. (TIF) [file pbio.1002443.s002.tif]

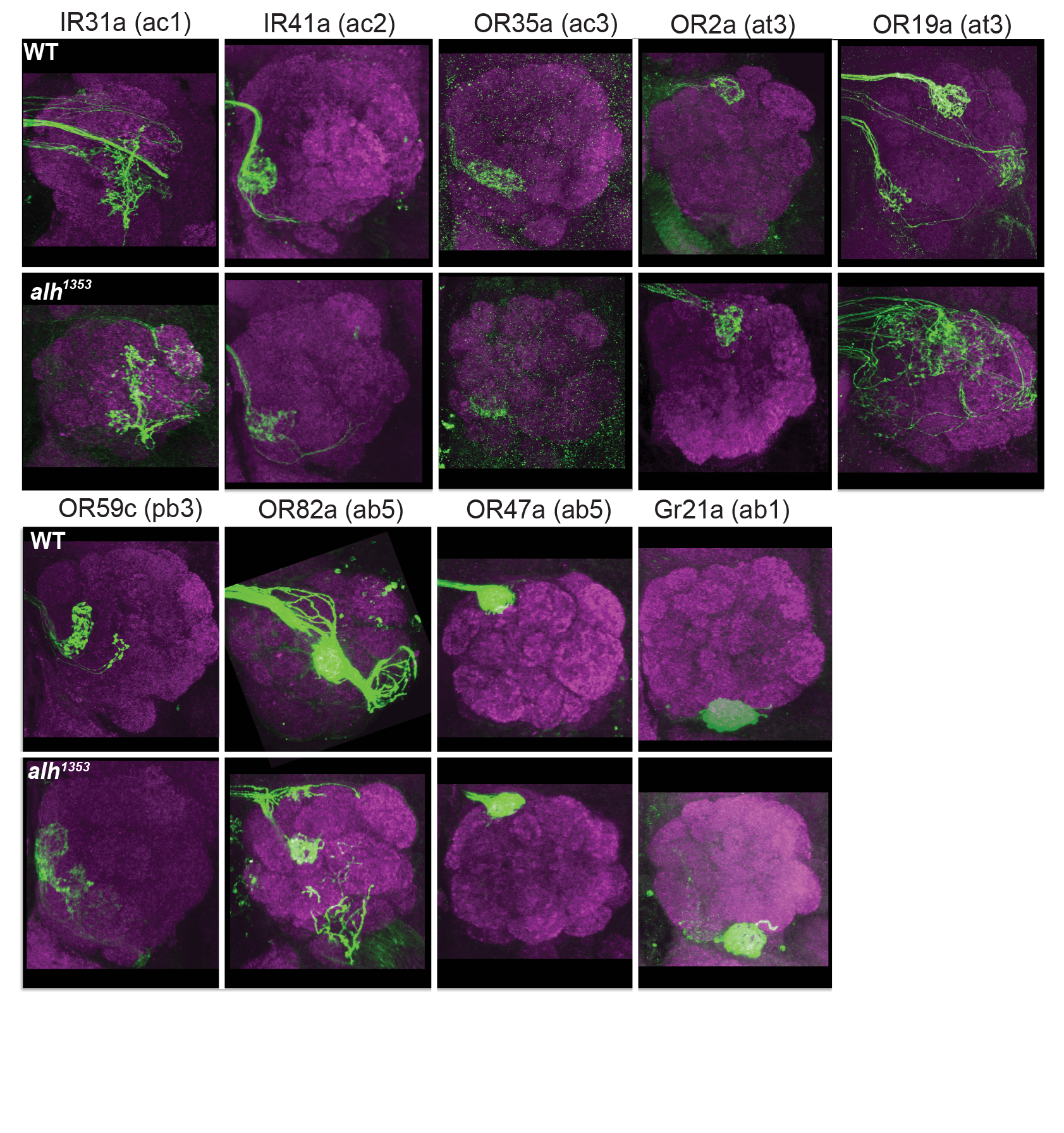

Supplement: S2 Fig — Connectivity of ORNs in wild type (top panels) and alh 1353 mutant (bottom panels) antennal lobes. (TIF) [file pbio.1002443.s003.tif]

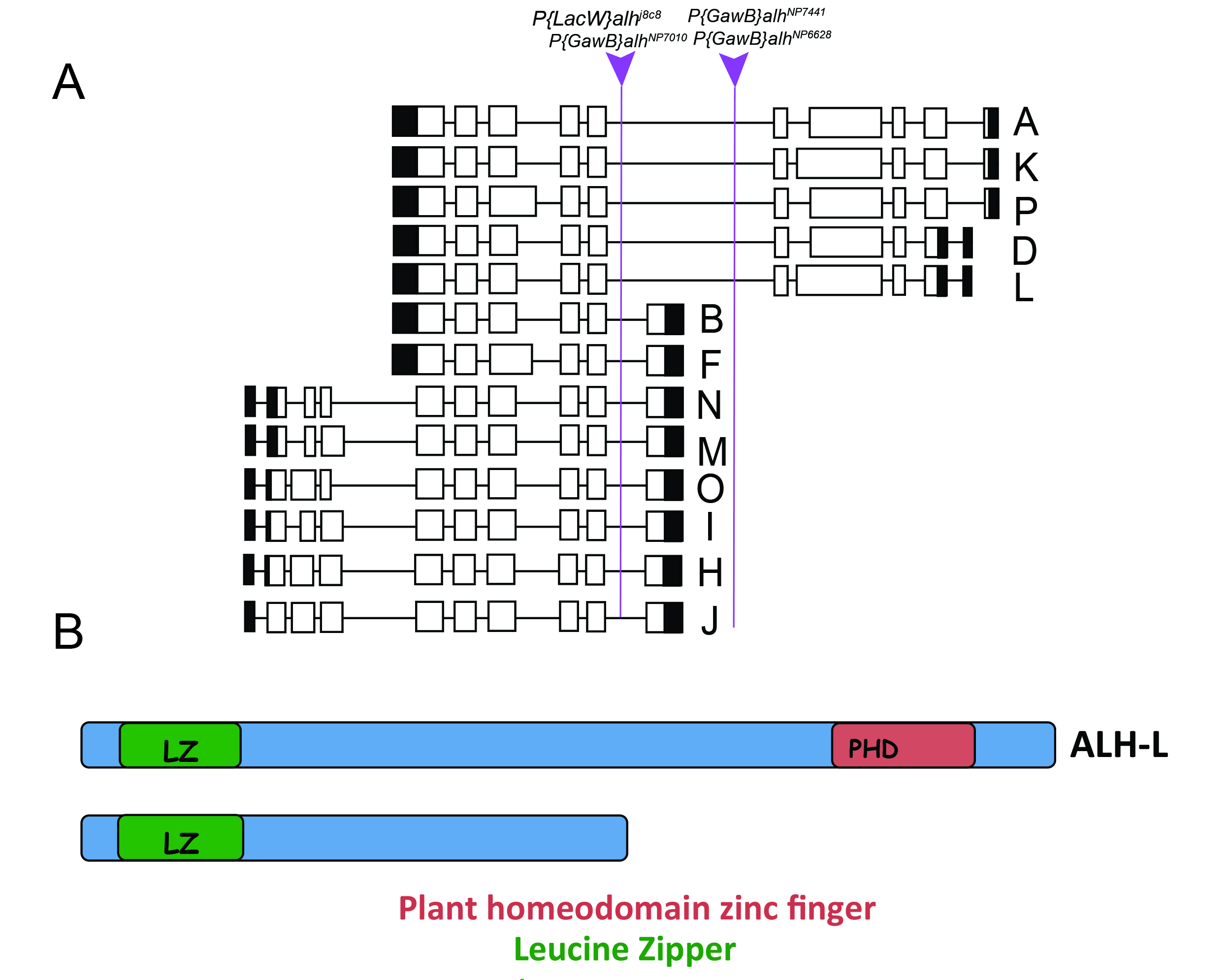

Supplement: S3 Fig — (A) alh splice isoforms (letters on the right denote the name of the isoform). The Alh j8c8 allele and the alh NP lines used in expression analysis are inserted in the first intron of the short isoforms, and the fifth intron of the long isoforms. (B) Major protein domains found in long and short Alh isoforms. (TIF) [file pbio.1002443.s004.tif]

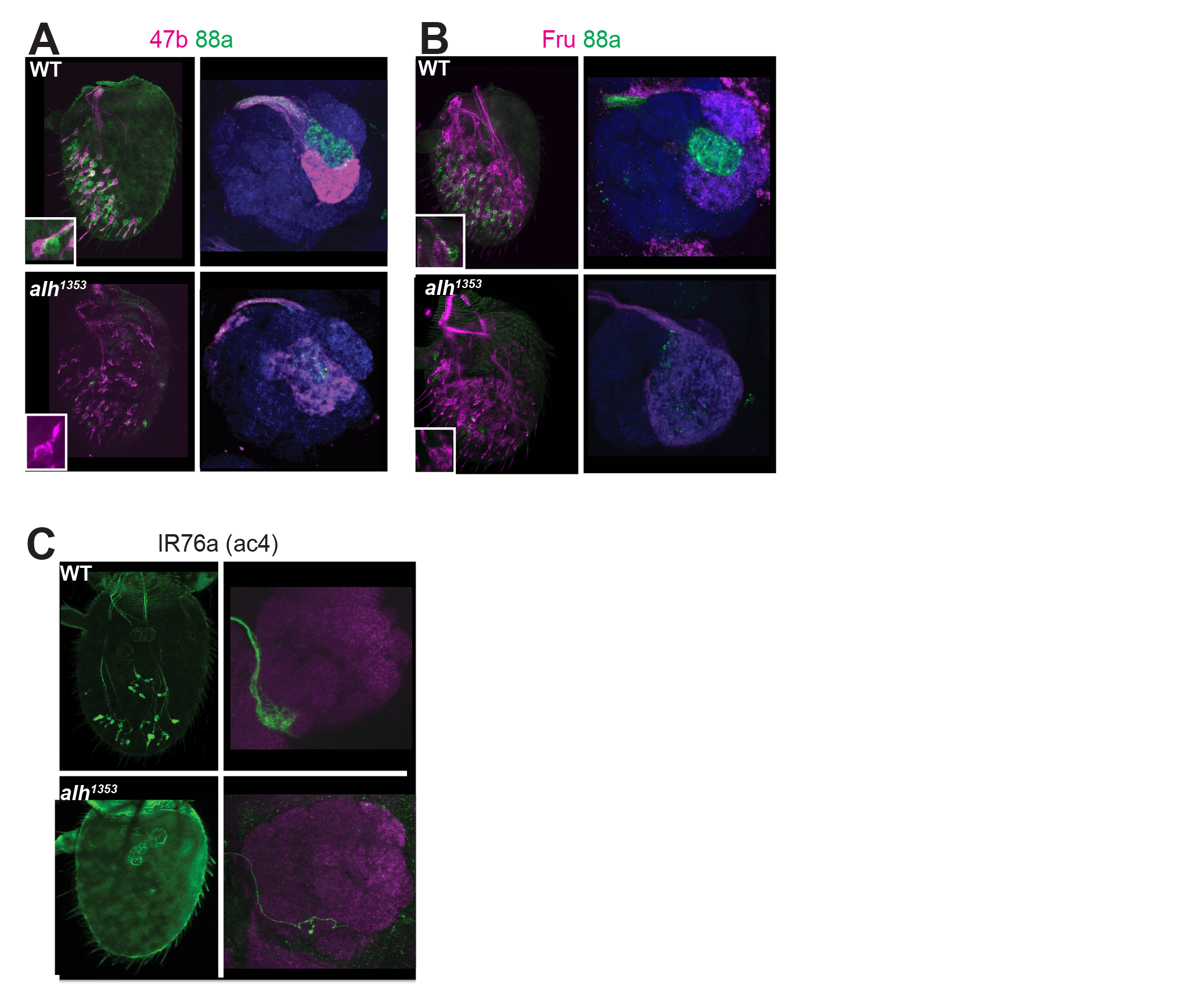

Supplement: S4 Fig — A) Double-labeling of Or47b and Or88a in WT and alh mutant antennae and antennal lobes. Or88a expression is not expanded to other ORNs in alh mutant antennae. B) Double-labeling of Or88a and fru in WT and alh mutant antennae and antennal lobes. Or88a expression does not overlap with fru expression in wild type or alh mutant antennae. C) Ir76a expression is decreased in alh mutant antennae and antennal lobes. (TIF) [file pbio.1002443.s005.tif]

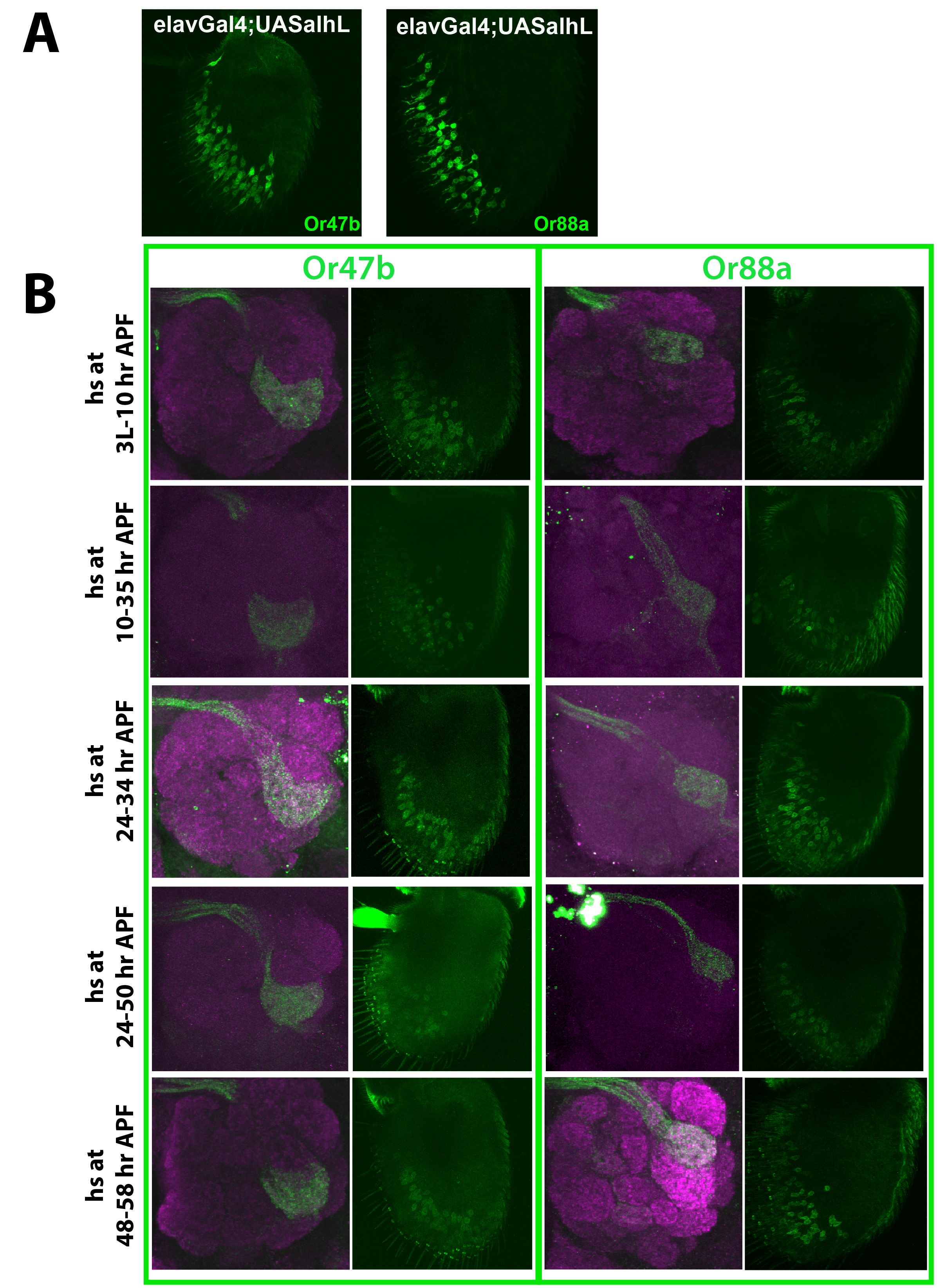

Supplement: S5 Fig — A) Expression of the long isoform of alh (alh-L) under the control of elav-GAL4 does not change the expression of either Or47b and or Or88a as assayed with direct fusion reporters in adult male antennae. (Expression of the short isoform of alh (alh-S) under the control of elav-GAL4 is lethal). B) Overexpression of the short isoform does not affect Or47b or Or88a expression. Flies were heat-shocked at 37°C for one hour during larval and pupal development at the indicated ages. (TIF) [file pbio.1002443.s006.tif]

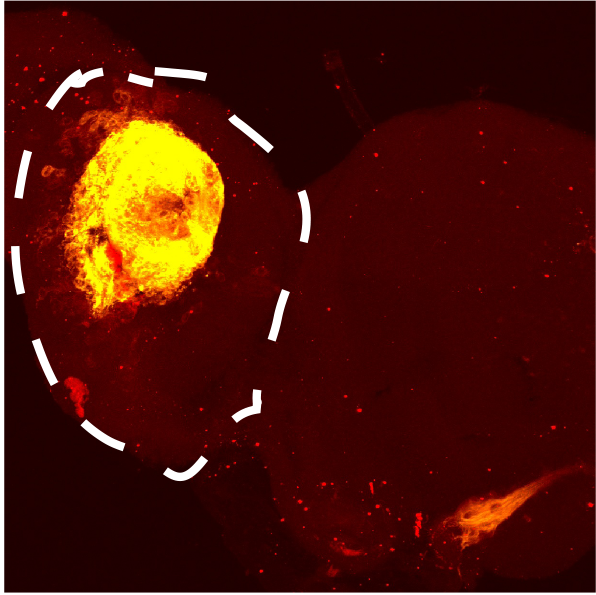

Supplement: S6 Fig — AlhGal4 expression in 3L larvae shows a spatial restriction to the center of the eye-antennal disc. (TIF) [file pbio.1002443.s007.tif]

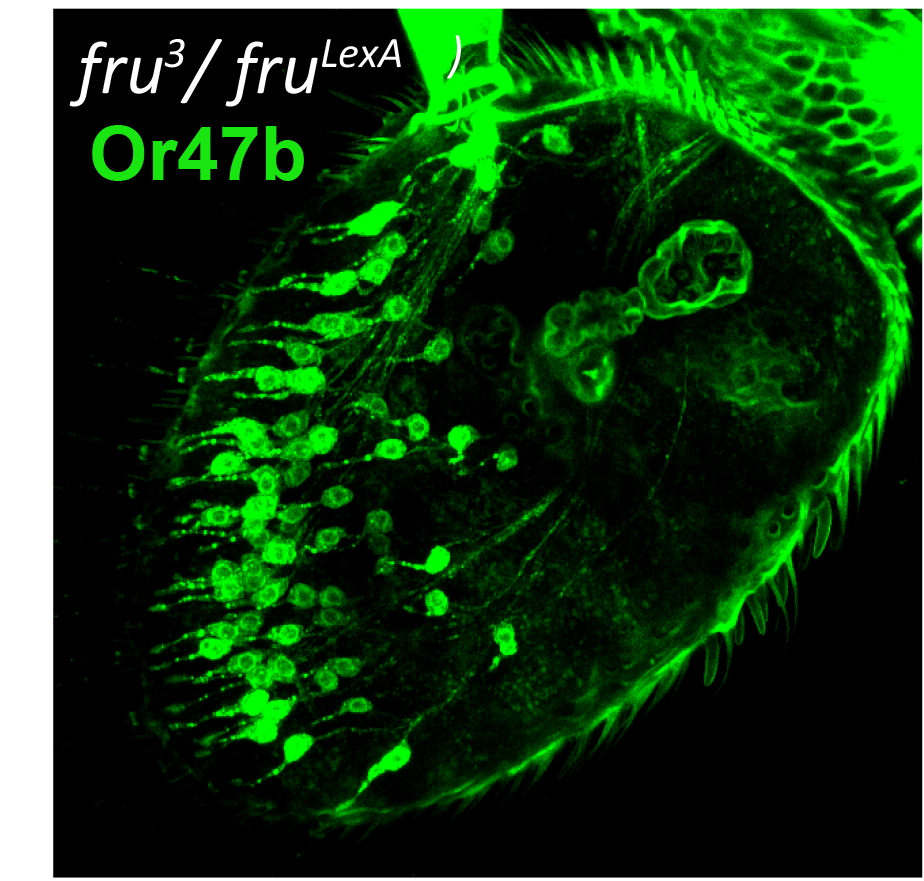

Supplement: S7 Fig — (TIF) [file pbio.1002443.s008.tif]

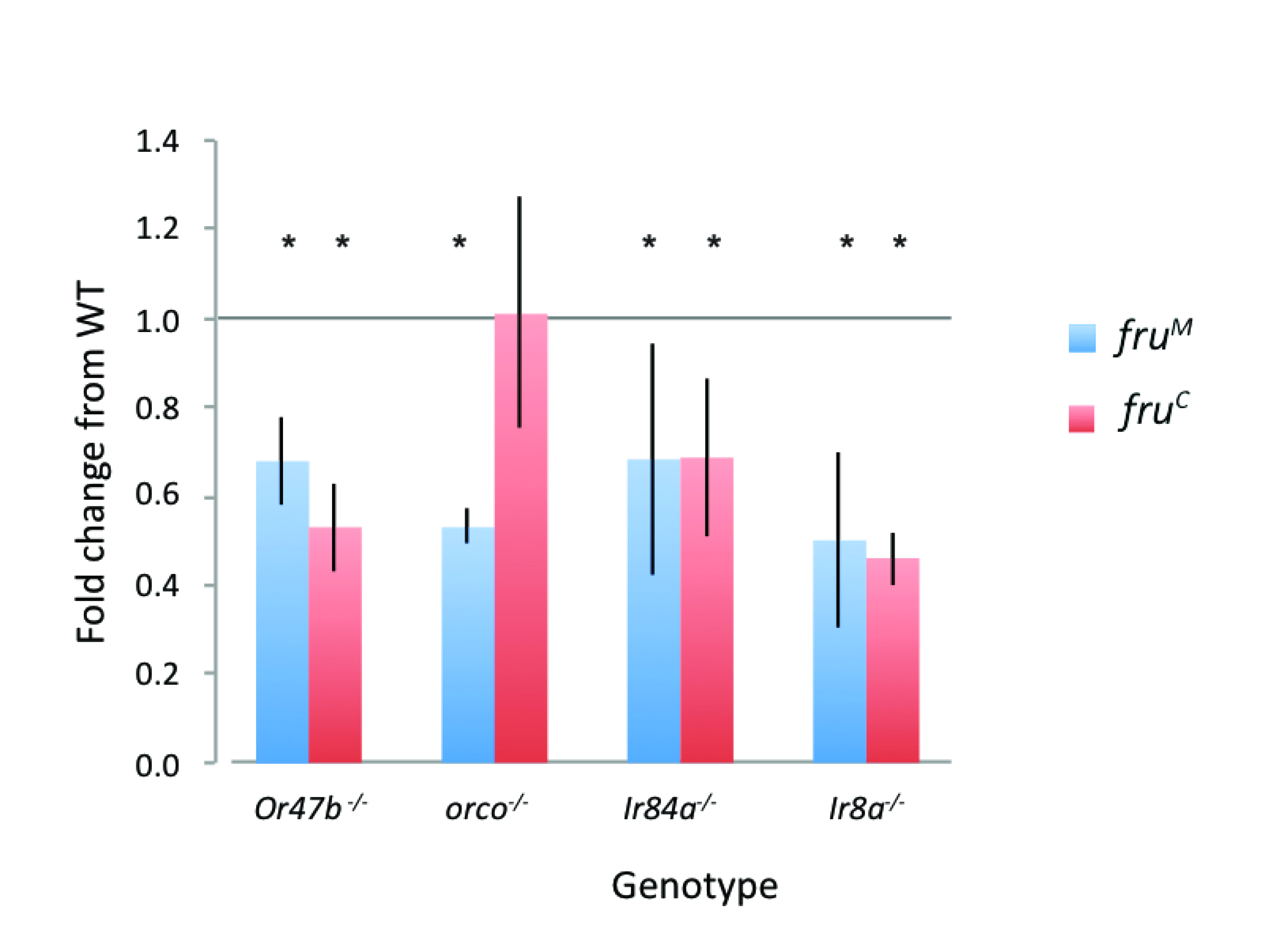

Supplement: S8 Fig — Data shown represents the fold change (normalized by the ΔΔCt method) in the expression of selected genes in the antenna as compared to w1118 control flies. A value of 1 indicates no change from control. Asterisks indicate p < .05 as measured by two-tailed t tests comparing Ct values of each genotype to controls. All fold change data may be found in the Supporting Information as S1 Data. (TIF) [file pbio.1002443.s009.tif]

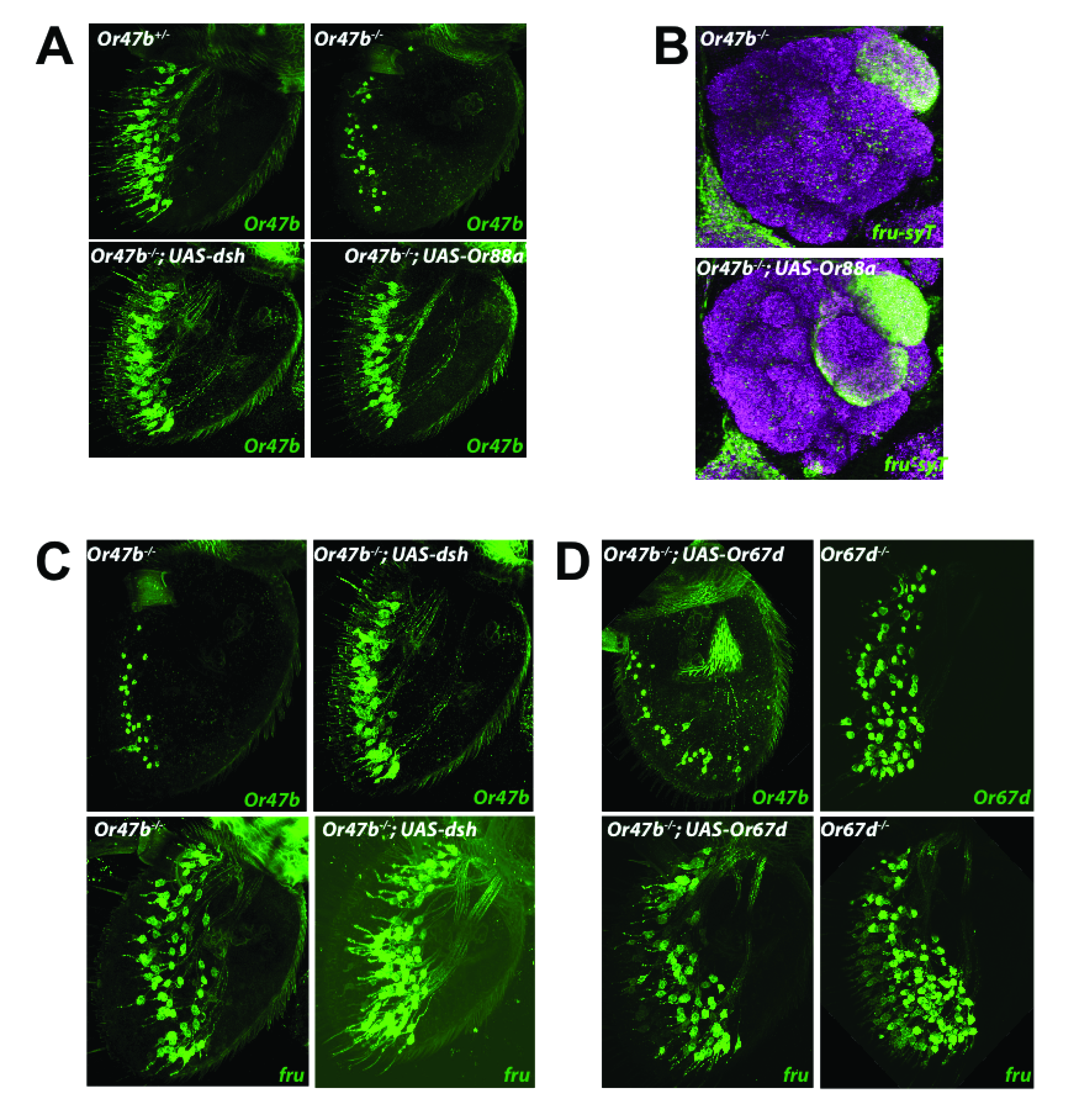

Supplement: S9 Fig — (A) Degeneration defects in Or47b mutants are rescued by UAS-dsh and UAS-Or88a overexpression using fru GAL4. (B) Expression of Or88a in Or47b mutants (bottom) partially rescues the loss of fru-positive ORN axon terminals (fru-syTGFP) in Or47b target glomerulus (top). (C) Loss of fru expression in or47b mutants is independent of neuronal degeneration. Degenerating Or47b ORN cell bodies are apparent by 14 d (left), which are rescued by overexpression of UAS-dsh (middle). However, despite the rescue of neuronal death, fru is still not expressed in or47b mutant antennae (right). (D) Or67d expression is not able to rescue the degeneration and fru expression defects in Or47b mutants (left panels). Or67d mutants do not have defects in fru expression and they do not degenerate by 14 d (right panels). (TIF) [file pbio.1002443.s010.tif]

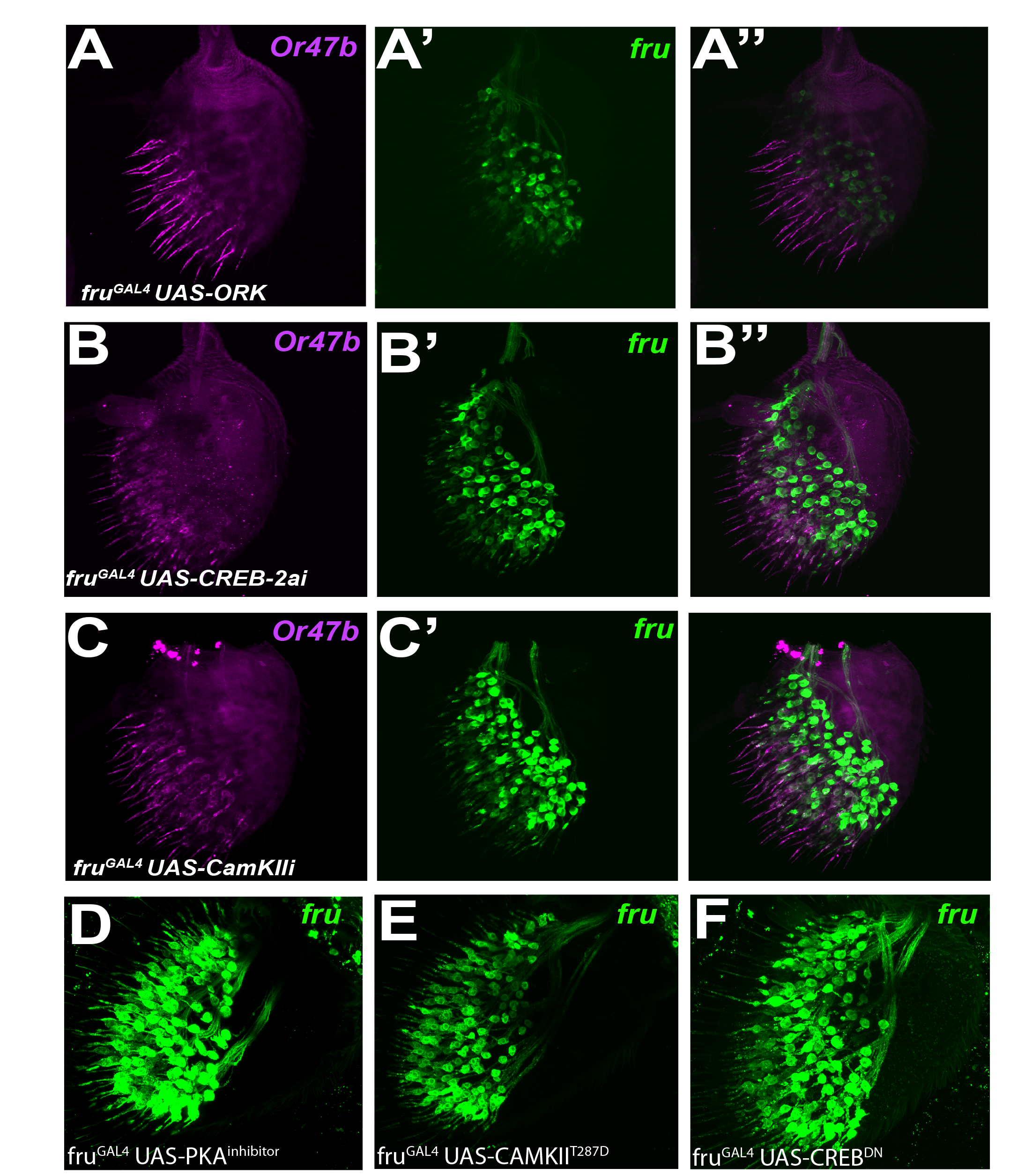

Supplement: S10 Fig — Fru expression is not affected by neuronal silencing using fru GAL4 driven UAS-ORK expression (A), or loss of Creb2a (B) and CamkII (C) function using fru GAL4-driven UAS-RNAi expression. Inhibition of PKA (D) and Creb (F) function by fru GAL4-driven UAS-PKA inhibitor and UAS-CREB DN expression, respectively, also does not affect fru expression. Constitutive activation of CamKII also does not have an effect (E). (TIF) [file pbio.1002443.s011.tif]

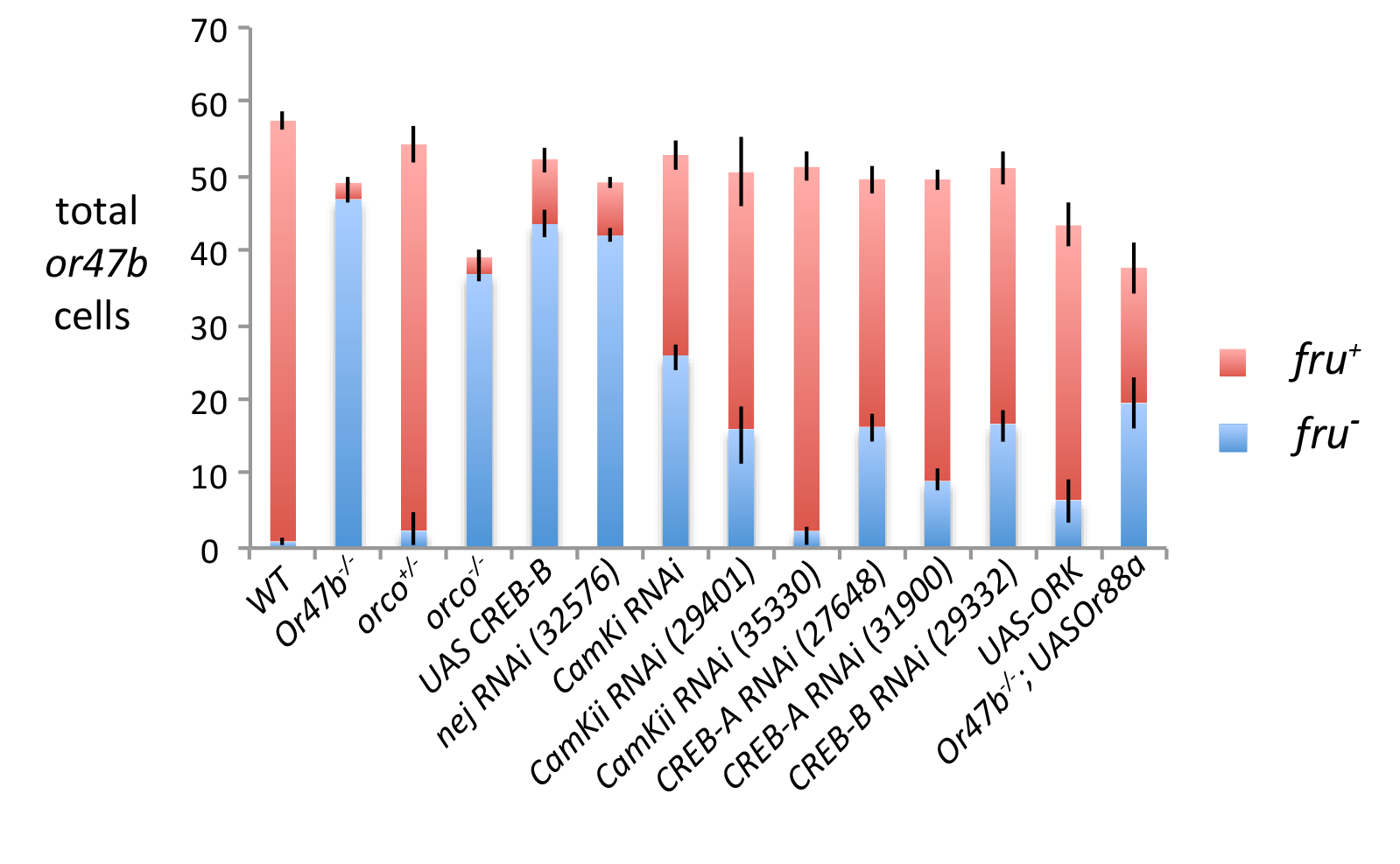

Supplement: S11 Fig — See Fig 8 in main text for significance. All raw count data may be found in the Supporting Information as S1 Data. (TIF) [file pbio.1002443.s012.tif]
